# Vitamin D and cognitive function: A Mendelian randomisation study

**DOI:** 10.1038/s41598-017-13189-3

**Published:** 2017-10-16

**Authors:** Jane Maddock, Ang Zhou, Alana Cavadino, Elżbieta Kuźma, Yanchun Bao, Melissa C. Smart, Kai-Uwe Saum, Ben Schöttker, Jorgen Engmann, Marie Kjærgaard, Ville Karhunen, Yiqiang Zhan, Terho Lehtimäki, Suvi P. Rovio, Liisa Byberg, Jari Lahti, Pedro Marques-Vidal, Abhijit Sen, Laura Perna, Henrik Schirmer, Archana Singh-Manoux, Juha Auvinen, Nina Hutri-Kähönen, Mika Kähönen, Lena Kilander, Katri Räikkönen, Håkan Melhus, Erik Ingelsson, Idris Guessous, Katja E Petrovic, Helena Schmidt, Reinhold Schmidt, Peter Vollenweider, Lars Lind, Johan G. Eriksson, Karl Michaëlsson, Olli T. Raitakari, Sara Hägg, Nancy L. Pedersen, Karl-Heinz Herzig, Marjo-Riitta Järvelin, Juha Veijola, Mika Kivimaki, Rolf Jorde, Hermann Brenner, Meena Kumari, Chris Power, David J. Llewellyn, Elina Hyppönen

**Affiliations:** 1 MRC Lifelong Health and Ageing at UCL, London, United Kingdom; 2 Population, Policy and Practice, UCL Great Ormond Street Institute of Child Health, London, United Kingdom; 3 Centre for Population Health Research, Sansom Institute, University of South Australia, Adelaide, Australia; 4 Wolfson Institute of Preventive Medicine, Queen Mary University of London, London, United Kingdom; 5 University of Exeter Medical School, Exeter, United Kingdom; 6 Institute for Social and Economic Research (ISER), University of Essex, Colchester, United Kingdom; 7 Division of Clinical Epidemiology and Aging Research, German Cancer Research Center (DKFZ), Heidelberg, Germany; 8 Network Aging Research (NAR), University of Heidelberg, Heidelberg, Germany; 9 Institute of Health Care and Social Sciences, FOM University, Essen, Germany; 10 Department of Epidemiology and Public Health, University College London, London, United Kingdom; 11 Division of Internal Medicine, University Hospital of North Norway, Tromsø, Norway; 12 Endocrinology Research Group, Department of Clinical Medicine, Faculty of Health Sciences, UiT The Arctic University of Norway, Tromsø, Norway; 13 Center for Life Course Health Research, University of Oulu, Oulu, Finland; 14 Oulu University Hospital, Oulu, Finland; 15 Department of Medical Epidemiology and Biostatistics, Karolinska Institutet, Stockholm, Sweden; 16 Department of Clinical Chemistry, Fimlab Laboratories and Faculty of Medicine and Life Sciences, University of Tampere, Tampere, Finland; 17 Research Centre of Applied and Preventive Cardiovascular Medicine, University of Turku, Turku, Finland; 18 Department of Surgical Sciences, Uppsala University, Uppsala, Sweden; 19 Department of Psychology and Logopedics, University of Helsinki, Helsinki, Finland; 20 Helsinki Collegium for Advanced Studies, University of Helsinki, Helsinki, Finland; 21 Department of Medicine, Internal Medicine, Lausanne University Hospital, Lausanne, Switzerland; 22 Department of Public Health and Nursing, Faculty of Medicine and Health Sciences, Norwegian University of Science and Technology (NTNU), Trondheim, Norway; 23 Department of Clinical Medicine, Faculty of Health Sciences, UiT The Arctic University of Norway, Tromsø, Norway; 24 Department of Cardiology, Akershus University Hospital, Lørenskog, Norway; 25 INSERM, U1018, Centre for Research in Epidemiology and Population Health, Hôpital Paul Brousse, Villejuif, France; 26 Department of Pediatrics, Tampere University Hospital and Faculty of Medicine and Life Sciences, University of Tampere, Tampere, Finland; 27 Department of Clinical Physiology, Tampere University Hospital and Faculty of Medicine and Life Sciences, University of Tampere, Tampere, Finland; 28 Department of Public Health and Caring Sciences, Uppsala University, Uppsala, Sweden; 29 Department of Medical Sciences, Uppsala University, Uppsala, Sweden; 30 Department of Medicine, Division of Cardiovascular Medicine, Stanford University School of Medicine, Stanford, California, USA; 31 Unit of Population Epidemiology, Division of Primary Care Medicine, Department of Community Medicine, Primary Care and Emergency Medicine, Geneva University Hospitals, Geneva, Switzerland; 32 Department of Epidemiology, Rollins School of Public Health, Emory University, Atlanta, Georgia, USA; 33 Department of Ambulatory Care and Community Medicine, University of Lausanne, Lausanne, Switzerland; 34 Division of General Neurology, Department of Neurology, General Hospital and Medical University of Graz, Graz, Austria; 35 Research Unit for Genetic Epidemiology, Institute of Molecular Biology and Biochemistry, Center of Molecular Medicine, Medical University of Graz, Graz, Austria; 36 Department of Neurology, Clinical Division of Neurogeriatrics, Medical University Graz, Graz, Austria; 37 Department of General Practice and Primary Health Care, University of Helsinki and Helsinki University Hospital, University of Helsinki, Helsinki, Finland; 38 Folkhälsan Research Center, Helsinki, Finland; 39 Department of Clinical Physiology and Nuclear Medicine, Turku University Hospital, Turku, Finland; 40 Research Unit of Biomedicine, University of Oulu, Oulu, Finland; 41 Biocenter Oulu, University of Oulu, Oulu, Finland; 42 Medical Research Center (MRC) and Oulu University Hospital, Oulu, Finland; 43 Department of Gastroenterology and Metabolism, Poznan University of Medical Sciences, Poznan, Poland; 44 Department of Epidemiology and Biostatistics, MRC–PHE Centre for Environment & Health, School of Public Health, Imperial College London, London, UK; 45 Research Unit of Clinical Neuroscience, Department of Psychiatry, University of Oulu, Oulu, Finland; 46 Department of Psychiatry, University Hospital of Oulu, Oulu, Finland; 47 Clinicum, University of Helsinki, Helsinki, Finland; 48 South Australian Health and Medical Research Institute, Adelaide, Australia

## Abstract

The causal nature of the association between hypovitaminosis D and poor cognitive function in mid- to later-life is uncertain. Using a Mendelian randomisation(MR) approach, we examined the causal relationship between 25(OH)D and cognitive function. Data came from 172,349 participants from 17 cohorts. *DHCR7*(rs12785878), *CYP2R1* rs12794714) and their combined *synthesis score* were chosen to proxy 25(OH)D. Cognitive tests were standardised into global and memory scores. Analyses were stratified by 25(OH)D tertiles, sex and age. Random effects meta-analyses assessed associations between 25(OH)D and cognitive function. Associations of serum 25(OH)D with global and memory-related cognitive function were non-linear (lower cognitive scores for both low and high 25(OH)D, *p*_curvature_ ≤ 0.006), with much of the curvature attributed to a single study. *DHCR7*, *CYP2R1*, and the *synthesis score* were associated with small reductions in 25(OH)D per vitamin D-decreasing allele. However, coefficients for associations with global or memory-related cognitive function were non-significant and in opposing directions for *DHCR7* and *CYP2R1*, with no overall association observed for the *synthesis score*. Coefficients for the *synthesis score* and global and memory cognition were similar when stratified by 25(OH)D tertiles, sex and age. We found no evidence for serum 25(OH)D concentration as a causal factor for cognitive performance in mid- to later life.

## Introduction

The profound effect of an ageing population is evidenced by estimates suggesting 65·7 million people worldwide will be affected by dementia by 2030, increasing to 115·4 million by 2050^1^. The latency period from the onset of symptoms to clinical diagnosis is typically very long and cognitive changes can be observed decades before diagnosis^2^. So, for the development of efficient primary prevention strategies, it is essential to identify risk factors that operate  at the early pre-clinical stage.

Hypovitaminosis D is hypothesised to be one such risk factor. Low 25-hydroxyvitamin D [25(OH)D] levels are prevalent in older individuals^3^, and adults with cognitive difficulties have been shown to have hypovitaminosis D^4–6^. There is a biologically plausible link between vitamin D and cognitive function. The vitamin D receptor (VDR), vitamin D metabolites and enzymes required for vitamin D activation have been found in the brain and central nervous system^7^. Additionally, experimental studies have demonstrated that active vitamin D may influence brain and neuron development^8^, and have neuroprotective potential and antioxidant effects^7^. Studies on VDR knockout mice have demonstrated that hypovitaminosis D may play a role in accelerated ageing, behavioural, social, motor and sensory deficits^9–12^, all of which can contribute to cognitive decline.

A number of observational studies have linked hypovitaminosis D with cognitive impairment and/or dementia^5,6,13,14^. In 2012, a meta-analysis including eight cross-sectional studies (*n* = 2,740) found that mean scores on the Mini-Mental State Examination (MMSE) were lower among individuals with <50 nmol/l compared with ≥50 nmol/l 25(OH)D^5^. The authors also demonstrated that 25(OH)D concentrations were on average 6·2 nmol/l lower in Alzheimer’s disease patients compared with controls (*n* = 502)^5^. Another meta-analysis in 2012, including five cross-sectional and two longitudinal studies (*n* = 7,688) suggested that the risk of cognitive impairment was doubled in participants with low vitamin D status compared to those with normal levels^6^. A systematic review in 2013 found that low vitamin D status was associated with worse cognitive function or a higher incidence of dementia in 72% of the 25 cross-sectional studies and 67% of the six prospective studies included^13^. In 2017, a meta-analysis including five longitudinal studies supported the hypothesis that 25(OH)D concentrations <25 nmol/l may contribute to the development of dementia^14^.

Results from randomised controlled trials (RCTs) have not been as promising^15–18^. However, null findings from these trials may be due to a number of reasons including: short follow-up time^16,18^, inclusion of younger participants^16^, inclusion of supraphysiological doses^18^, vitamin D being used in combination with other substances^15,17^, in low doses^17^ or, high baseline 25(OH)D status^15,16^.

In light of these findings, in 2014 a group of international experts came to the consensus that hypovitaminosis D should be considered a risk factor for cognitive decline and dementia as it may change the clinical presentation of dementia due to accompanying comorbidities, but that 25(OH)D should not be used as a diagnostic or prognostic biomarker^19^. The authors concluded that vitamin D supplementation should be part of the care management of older adults with cognitive disorders^19^. However, whether vitamin D plays a causal role in cognitive decline directly or through its impact on comorbidities, or whether it is a consequence of cognitive decline remains unclear.

Determining the nature of the true relationship between vitamin D and cognitive function is challenging due to study design issues. For instance, even if well-conducted, observational studies may not capture all unmeasured confounding and there is a possibility of reverse causality^20^. RCTs are the gold standard approach for inferring a causal association, but they also have their limitations^20^. Mendelian Randomisation (MR) is an approach that uses a genetic variant, which is associated with the exposure of interest, to estimate the causal relationship between an exposure and outcome^20^. This method can help to overcome some limitations of observational studies as it relies on the random assignment of genetic variants from parents to offspring to reduce the possibility of confounding^20^. Furthermore, since the genetic variant is established at conception, the possibility of reverse causality is minimised^20^. If hypovitaminosis D is causally related to worse cognitive function, the genetic variant associated with vitamin D status, should be associated with cognitive function. Using an MR approach, a recent study provided some support for a beneficial role of 25(OH)D in reducing the risk of dementia^21^.

We conducted a study to examine the causal nature of the association between vitamin D status, as measured by 25(OH)D, and cognitive function in mid- to later-life using a MR approach.

## Methods

### Participants

Information came from 17 cohorts: 1958 British birth cohort (1958BC); Austria Stroke Prevention Study (ASPS); The CoLaus Study (CoLaus); English Longitudinal Study of Ageing (ELSA); Epidemiologic study assessing prevention, early detection, and treatment of chronic diseases among older adults (ESTHER); Helsinki Birth Cohort Study (HBCS); Health and Retirement Study (HRS); Northern Finland Birth Cohort 1966 (NFBC1966); The Prospective Investigation of the Vasculature in Uppsala Seniors (PIVUS); Swedish Twin Registry (STR); The Tromsø Study (Tromsø); TwinGene; UK Biobank; The UK Household Longitudinal Study (*Understanding Society*, UKHLS); Uppsala Longitudinal Study of Adult Men (ULSAM); Whitehall II (WII); and Young Finns Study (YFS). In all studies, data were restricted to White/European participants with genetic and cognitive data (*n* = 172,349, Table 1). Information on 25(OH)D concentrations was available in nine studies (*n* = 26,856). All participants provided informed consent and ethical approval was granted by local research ethics committees. An expanded description is provided in supplementary text.

**Table 1:** Participant characteristics.

	**Total**	**Males**	**Age, yrs**	**Age** ≥ **65yrs**	**25(OH)D, nmol/l**	***CYP2R1*** **, MAF**	***DHCR7*** **, MAF**
	**N** = **172,349**	**(%)**	**(median, IQR)**	**(%)**	**(median, IQR)**	**(%)**	**(%)**
***Studies with 25(OH)D (N*** = ***28,070***)							
1958BC	5,633	49·1	50 (NA)	0	57·0 (33·9)	43·0	22·2
CoLaus	875	45·1	70 (6)	100	47·3 (33·6)	47·1	27·9
ESTHER	8,080	43·0*	74 (4)*	100*	45·1 (27·1)	46·2	25·7
HBCS	1,059	59·1	67·6 (3·6)	91·8	61 (24)	38·5	38·0
NFBC66	3,488	43·5	46·5 (0·9)	0	50·2 (20·8)	40·8	38·9
PIVUS	891	50·3	70·1 (0·2)	100	56 (26·5)	39·9	35·0
Tromsø	4,766	55·4	69 (11)	76·8	56·5 (25·9)	41·2	38·6
ULSAM	1,118	100	71 (0·9)	100	68·2 (24·7)	39·4	33·4
YFS	2,160	45·1	43 (9)	0	57 (25)	38·2	40·4
***Studies without 25(OH)D (N*** = ***144,279***)							
ASPS	780	43·3	64.7 (11)	49·2	NA	43·7	29·3
ELSA	5,382	45·6	65 (15)	50·6	NA	43·2	22·4
HRS	9,930	41·4	68 (15)	62	NA	43·8	27·0
STR	969	44·7	72.1 (10·1)	77·2	NA	38·8	30·0
TwinGene	2,362	51·1	69 (6)	100	NA	40·0	33·2
UKBiobank	111,936	47·5	58 (12)	19·7	NA	42·2	21·0
UKHLS	8,577	43·7	54 (24)	28·0	NA	42·4	21·6
WII	4,343	76·2	59·7 (10)	28·7	NA	42·5	22·7

IQR: Interquartile range; MAF: minor allele frequency.

1958BC: 1958 British birth cohort;

COLAUS: The Colaus study;

ESTHER: Epidemiologische Studie zu Chancen der Verhütung, Früherkennung und optimierten Therapie chronischer Erkrankungen in der älteren Bevölkerung;

HBCS: Helsinki Birth Cohort Study;

NFBC66: Northern Finland birth cohort 1966;

PIVUS: The Prospective Investigation of the Vasculature in Uppsala Seniors;

TROMSO: The Tromsø Study;

ULSAM: Uppsala Longitudinal Study of Adult Men;

YFS: Young Finns;

ASPS: Austria Stroke Prevention Study;

ELSA: English Longitudinal Study of Ageing;

HRS: Health and Retirement Study;

STR: Swedish Twin Registry;

TwinGene: Swedish Twin Registry;

UKBiobank: UK Biobank;

UKHLS: The UK Household Longitudinal Study (Understanding Society);

WII: Whitehall II

* N’s based on participants with cognitive data.

### Genetic variants

We used two single nucleotide polymorphisms (SNPs) based on their demonstrated associations with 25(OH)D concentrations: rs12785878 (vitamin D-decreasing allele, G), located near gene coding 7-dehydrocholesterol reductase (*DHCR7*), and rs12794714 (vitamin D-decreasing allele, A) near 25-hydroxylase (*CYP2R1*)^22,23^. When these SNPs were not accessible, proxy SNPs in perfect linkage disequilibrium were used (Supplementary Table 1). The assumptions for the use of these SNPs to proxy vitamin D status in MR studies have been assessed in a previous study using data from 1958BC^24^. Data on *DHCR7* and *CYP2R1* were available in all cohorts. For analysis purposes, both *DHCR7* and *CYP2R1* genotypes were coded as 0-2 depending on presence of alleles associated with decreasing 25(OH)D concentrations, where homozygous genotypes were coded as 2. A score was created by summing *DHCR7* and *CYP2R1* on the basis of their effect alleles^24^. The score is referred to as the *synthesis score* since it contains the SNPs involved in the synthesis of 25(OH)D^24^. The few participants with 3 or 4 25(OH)D-decreasing alleles (ranging from 9·6% in 1958BC to 17·4% in Tromsø) were grouped. Genotyping techniques are described in supplementary materials. Quality checks of each SNP including minor allele frequencies and Hardy Weinberg equilibrium are reported in Supplementary Table 1. Minor allele frequencies were compared with HapMap data and were found to be approximately similar.

### 25-Hydroxyvitamin D

25(OH)D concentrations were available for nine studies, with details for measurement methods provided in the supplementary text. In order to examine analyses stratified by 25(OH)D concentrations, sex and study specific 25(OH)D tertiles(T) were created (Supplementary Table 2). 25(OH)D was found to be skewed, therefore natural log (ln) transformation was applied to approximate a normal distribution when 25(OH)D was the outcome in analyses.

### Global and memory cognitive function

Details of cognitive tests in each cohort, grouped to represent global and/or memory cognitive function, can be found in supplementary materials. Each test was standardised to produce a mean of zero and a standard deviation of one. To obtain a summary score for global/memory cognitive function, relevant tests were summed and re-standardised. ULSAM and CoLaus had information on global cognitive function only.

### Covariates

Results in all studies were adjusted for sex, age (in years), month of blood collection for 25(OH)D, and, education and depressive symptoms which were considered potential confounders *a priori*. A description of how education and depressive symptoms were measured in each cohort is outlined in supplementary materials.

### Statistical analyses

Within each study, linear regression models were used to assess the following: 1) phenotypic associations i.e. serum 25(OH)D and cognitive function adjusted for age, sex, month of 25(OH)D blood collection, educational attainment and depressive symptoms where possible; 2) associations between SNPs/*synthesis score* and cognitive function adjusted for age, sex, education, depressive symptoms and principle components (to account for population stratification) where specified in supplementary materials. The presence of non-linear phenotypic associations were assessed by including a quadratic term of 25(OH)D in the regression model. Interaction by age and sex was also assessed.

Results from within-study analyses were combined using random effects meta-analyses. Analyses were stratified by 25(OH)D-tertiles, sex and age (<65 years vs. ≥65 years). I-square tests were used to indicate heterogeneity between cohorts.

Meta-regression was used to examine heterogeneity between the cohorts using results from the meta-analysed phenotypic analyses. Study characteristics that were hypothesised *a priori* to affect the association included sex, age (<65 years vs. ≥65 years), vitamin D assay (mass spectrometry or immunoassay) and country region [categorised as UK, Nordic (Finland, Sweden, Norway), central Europe (Austria, Germany, Switzerland) and the US].

To examine the strength and suitability of the SNPs/*synthesis score* as instruments for MR studies, associations between the SNPs/*synthesis score* and ln25(OH)D (adjusted for age, sex, month of 25(OH)D collection and study-specific components where specified) were examined, and F-statistics were calculated. The F-statistic was approximated from the proportion of variation in the model (R^2^) assessing 25(OH)D which is explained by the SNPs/*synthesis score* [F-stat = (R^2^*(*n* − 2))/(1 − R^2^)]. The adjusted R^2^ in each cohort was weighted by the sample size of each cohort. The F-statistic should ideally be greater than ten in order for an instrument to be considered strong enough to use^25^.

Participants from UK Biobank were used to investigate cognitive domain-specific effects (using standardised fluid intelligence, pairs matching and reaction time tests) of the SNPs/*synthesis score*.

Finally, a power calculation was conducted to determine the smallest effect size that our study able to detect at a two-sided alpha level of 0·05 and at a power of 0.80. Power analysis was performed using Quanto 1.2 (University of Southern California, USA).

Meta-analyses were conducted at the Centre for Population Health Research (University of South Australia) using STATA version 14.

## Results

A total of 172,349 participants had complete data for the SNPs and completed at least one cognitive test. Participants from eight cohorts had no information on 25(OH)D concentrations, while two studies had no information on memory-related cognitive function. Basic characteristics of all studies are presented in Table 1.

### Phenotypic association between serum 25(OH)D and cognitive function

In meta-analyses of the nine eligible studies, there was no evidence of interaction by sex or age on phenotypic associations (*p* ≥ 0·06, Supplementary Table 3). There was evidence of a non-linear relationship between serum 25(OH)D and cognitive function, *p* ≤ 0·006 (Supplementary Table 3) after adjustment for age, sex, month of blood collection, educational attainment and depressive symptoms. However, this non-linear association was driven by a single study and weakened following its’ exclusion from the meta-analyses (Table 2). When stratified by 25(OH)D tertiles, participants in T2 and T3 had higher scores in global cognition compared with those in T1 (0·05 SD, 95%CI 0·01, 0·09; p = 0·02 and 0·07 SD, −0·01, −0·15; p = 0·07, respectively), while no clear differences were not seen for memory cognition (P ≥ 0·11 for both comparisons). There were no overall differences in phenotypic associations by sex, country or vitamin D assay, while associations appeared somewhat stronger among those aged 65 years or above compared to younger participants (Supplementary Table 4).

**Table 2:** Association of sex-specific 25(OH)D tertiles with cognition.

	Global Cognition					Memory Cognition				
	N	Beta (95% C.I.)	p	I^2^(%)	p_hetero_	n	Beta (95% C.I.)	p	I^2^(%)	p_hetero_
**25(OH)D T1**	4,961	Reference				44,482	Reference			
**25(OH)D T2**	5,269	0·05 (0·01, 0·09)	0·02	17·48	0·29	44,772	0·04 (−0·01, 0·09)	00·11	34·29	0·17
**25(OH)D T3**	5,293	0·07 (−0·01, 0·15)	0·07	74·05	<0·001	44,827	0·02 (−0·07, 0·11)	0·64	76·84	<0·001
		p_trend_ = 0·15					p_trend_ = 0·89			
		p^*^_curvature_ = 0·001					p^┼^_curvature_ = 0·01			

^*^excluding 1958BC, p_curvature_ = 0·04; ^┼^excluding 1958BC, p_curvature_ = 0·16.

### Mendelian randomisation: association between SNPs/synthesis score and cognitive function

**Figure 1:**
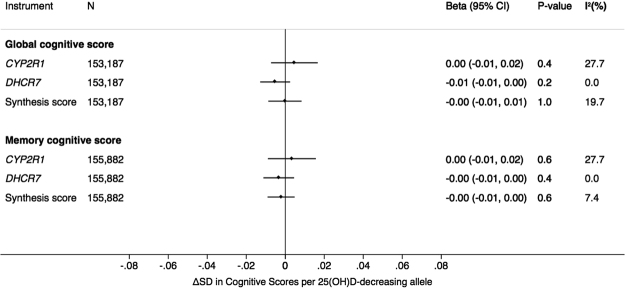
Association of *CYP2R1*, *DHCR7* and *synthesis score* with global and memory cognition.

There were no associations between 25(OH)D-related SNPs/*synthesis score* with global or memory cognition (Fig. 1, Supplementary Table 5). Furthermore, there was no evidence for an association between SNPs/*synthesis score* with either cognitive measure stratifying by sex, age and 25(OH)D tertiles (Supplementary Figure 1). Genetic associations with cognitive function did not vary by age, sex or country (Supplementary Table 4).

Information for pairs matching (*n* = 110,545), reaction time (*n* = 109,911), reasoning (*n* = 35,603), and prospective memory (*n* = 36,311) was available for the UK Biobank. We conducted domain specific analyses against all the four outcomes, but observed no associations with the SNPs/*synthesis score* (Supplementary Figure 2).

### Instrument validation: association between SNPs/synthesis score and 25(OH)D

**Figure 2:**
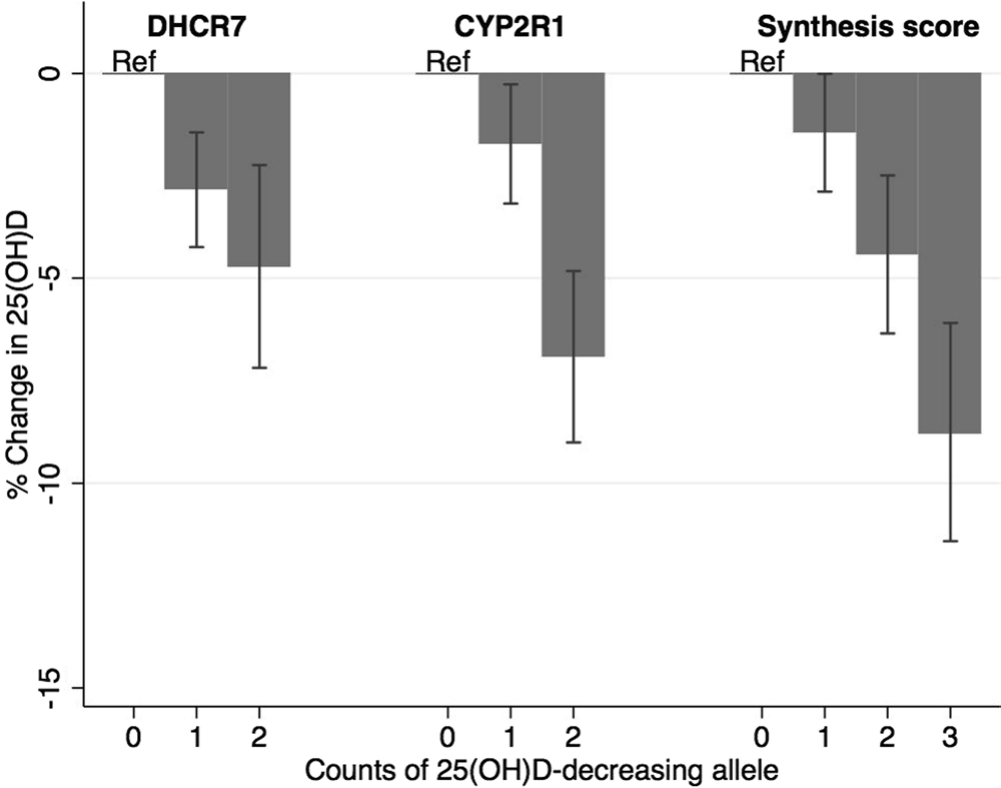
Association of *CYP2R1*, *DHCR7* and *synthesis score* with 25-hydroxyvitamin D (25(OH)D).

The SNPs/*synthesis score* were associated with 25(OH)D (Fig. 2, Supplementary Table 6). 25(OH)D concentrations were 2·7% (95% CI 0·7% to 4·1%), 3·3% (95% CI 0·5% to 4·3%), and 3·1% (95% CI 0·5% to 4·0%) lower per vitamin D-decreasing allele for *DHCR7*, *CYP2R1* and the *synthesis score* respectively. The weighted F-statistic was 54·13 (R^2^ = 0.003), 71·69 (R^2^ = 0.004) and 113·50 (R^2^ = 0.006) for *DHCR7*, *CYP2R1* and the *synthesis score* respectively. Since these F-statistics are >10, the SNPs/*synthesis score* can be considered strong proxies for 25(OH)D in MR analyses^25^.

### Power calculation

With a sample size of 153,187, a power of 0·80, and a two-sided alpha level of 0·05, we are sufficiently powered to detect a 0·0125 SD change in the cognitive score per vitamin D-decreasing allele. For the smallest stratum with *n* = 4,957, our study is powered to detect an effect size of 0·058 SD or larger (Supplementary Table 7). Converted to the reflect the association between serum 25(OH)D and a standardised cognitive outcome, this corresponds to a 0.09 SD and 0.51 SD difference by 1 SD change in log 25(OH)D for the full and minimum sample, respectively.

## Discussion

Using data from 17 studies with information for up to 172,349 participants, we failed to find any evidence for a causal association between vitamin D and cognitive function, with sub-group analyses stratifying by age, sex and 25(OH)D tertiles providing a consistent lack of evidence for causality. These findings suggest that the non-linear phenotypic association between serum 25(OH)D and cognitive function (where cognitive scores were lower for both low and high 25(OH)D concentrations), which was also confirmed in our datasets, may be due to reverse causation or confounding.

There are a limited number of RCTs assessing the effect of vitamin D supplementation on cognitive function^15–18^. In line with our study, these have provided little evidence for causality. In particular, our MR findings were supportive of results from the biggest RCT to date which was carried out among older women (≥65 years, *n* = 4,143) participating in the Women’s Health Initiative^15^. In that RCT, over a mean follow-up of 7·8 years, there was no effect of supplementation with 400 IU/day vitamin D3 and 1,000 mg/day calcium on cognitive impairment. However, it has been argued that the inclusion of calcium, which can be harmful for the brain, may have weakened the result, and baseline concentrations of 25(OH)D among participants may have been adequate to meet their cognitive requirements^26^. Two pre-post studies have been conducted^27,28^. One found no effect of four weeks of vitamin D2 supplementation on cognitive function among institutionalised older adults^27^. In contrast with our findings, the other study demonstrated an improvement in the cognitive performance among 20 older adults after 16 months of 800 IU/day vitamin D3 supplementation compared with controls (*n* = 24)^28^. However, the non-random pre-post design of these studies is a limitation as potential unmeasured confounding cannot be ruled out.

One earlier genetic study suggested a beneficial effect of 25(OH)D on reducing the risk of Alzheimer’s disease^21^. However, in this study the beneficial association with Alzheimer’s disease was due to a significant association with a variant in the *GC* gene coding variations in the vitamin D binding protein, while in line with our study, no associations were seen for *DHCR7*, or *CYP2R1*. As we have described earlier, while associated with 25(OH)D concentrations, *GC* is not suitable for the use as its’ proxy marker in Mendelian randomisation analyses, given likely influences on bioavailability of 25(OH)D^29^. Indeed, the success of an MR study relies upon the ability of the genetic variant to accurately proxy the exposure of interest^20^. In line with previous studies^22–24^ we used two variants which have been consistently associated with circulating 25(OH)D concentrations. Both variants are located upstream of the 25(OH)D metabolite, with *DHCR7* influencing substrate availability and *CYP2R1* coding the 25-OH-hydroxylate. Analyses stratifying by 25(OH)D concentrations suggested that the association between *CYP2R1* and 25(OH)D is restricted to participants with the highest group. While this could suggest that the *CYP2R1* variant has a rate-limiting effect on 25(OH)D synthesis for individuals with the high 25(OH)D concentrations, it is also possible that the apparent difference is due to the wide range of 25(OH)D concentrations for individuals in the highest tertile in this study. Stratification by 25(OH)D tertiles could also have led to collider bias, i.e. when the association of two variables (i.e. genetic variant and cognitive function), changes upon conditioning on a third variable (i.e. 25(OH)D), when this third variable is affected by the other two. Therefore, cautious interpretation is required when stratifying MR analyses by 25(OH)D.

Interpreting the association between vitamin D and cognitive function is complicated. Dementia is often accompanied by a range of other chronic diseases/disorders, where cognitive decline may enhance chronic disease and vice-versa^19^. Vitamin D supplementation has been shown to reduce mortality risk^30^. Hypovitaminosis D has been associated with a number of conditions including osteoporosis, vascular disease and reduced olfactory function^31^, which can precipitate the progression of dementia. Therefore it is plausible that the potential cognitive benefits of vitamin D identified in observational studies may be mediated by improvements in accompanying chronic diseases.

Results should be interpreted with limitations in mind. It has been suggested that there are sensitive periods i.e. foetal development, growth and senescence during which vitamin D is of particular significance to neurocognition^19^. Our study looked at effects on cognitive function in mid- to later-life, and while we found no evidence stratifying by age group (i.e. <65 versus ≥65 years), we could not assess the association in younger or very old individuals. Nevertheless, the use of genetic variants to proxy 25(OH)D status assumes that we have represented lifetime 25(OH)D status^20^. Generalisability of the study results is restricted to caucasian populations. Cognition was assessed using different tests between the cohorts therefore a composite measure of global cognitive function was used to obtain a more uniform representation of cognitive function; this may have masked some domain specific effects. Since our previous study emphasised the role of vitamin D in cognitive function, a separate memory function score was created to account for any discriminating effects of vitamin D^32^. A recent study suggested that vitamin D may be associated with speed of processing and executive functioning^33^, we found no evidence for domain-specific effects using data from UK Biobank. MR studies require large sample sizes partly due to the very small amount of variation in the exposure explained by genetic instruments^20^. According to the power calculation, our study was sufficiently powered to detect relatively small effects. Nevertheless, it should be acknowledged that our analyses may have been underpowered to detect small causal effects operating at the extremes of 25(OH)D distribution.

## Conclusion

We found no evidence for a causal association between 25(OH)D concentrations and cognitive performance in mid- to later-life.

## Electronic supplementary material

Supplementary material
