# Supplementary material for: Vitamin D and cognitive function: A Mendelian randomisation study
Source: Sci Rep. 2017 Oct 16;7:13230. doi: 10.1038/s41598-017-13189-3 (PMC5643555; doi:10.1038/s41598-017-13189-3)
Supplement: Supplementary file 1 — Supplementary material [file 41598_2017_13189_MOESM1_ESM.pdf]

Supplementary Material

Vitamin D and cognitive function: A Mendelian randomisation study

Authors: Jane Maddock PhD[1,2*], Ang Zhou PhD[3], Alana Cavadino MSc[2,4], Elżbieta Kuźma PhD[5], Yanchun Bao PhD[6], Melissa C Smart PhD[6], Kai-Uwe Saum PhD[7], Ben Schöttker PhD [7,8,9], Jorgen Engmann MSc[10], Marie Kjærgaard PhD[11,12], Ville Karhunen MSc[13,14], Yiqiang Zhan MD[15], Terho Lehtimäki PhD [16], Suvi P. Rovio PhD[17], Liisa Byberg PhD[18], Jari Lahti PhD[19,20], Pedro Marques-Vidal PhD[21], Abhijit Sen PhD[22], Laura Perna PhD[7], Henrik Schirmer PhD[23,24], Archana Singh-Manoux PhD[10,25], Juha Auvinen PhD[13,14], Nina Hutri-Kähönen PhD[26], Mika Kähönen PhD[27], Lena Kilander MD PhD[28], Katri Räikkönen PhD[19], Håkan Melhus MD PhD[29], Erik Ingelsson MD PhD[29,30], Idris Guessous PhD[31,32,33], Katja E Petrovic PhD[34], Helena Schmidt MD PhD[35], Reinhold Schmidt MD[36], Peter Vollenweider MD[21], Lars Lind MD PhD[29], Johan G Eriksson DMSc [37,38], Karl Michaëlsson MD PhD [18], Olli T. Raitakari PhD [17,39], Sara Hägg PhD[15], Nancy L. Pedersen PhD[15], Karl-Heinz Herzig PhD[40,41,42,43], Marjo-Riitta Järvelin PhD[13,44], Juha Veijola MD[42,45,46], Mika Kivimaki PhD [10,47], Rolf Jorde PhD [11,12], Hermann Brenner MD[7,8], Meena Kumari PhD[6,10], Chris Power PhD[2], David J. Llewellyn PhD[5], Elina Hyppönen PhD[3,2,48*]

[1]MRC Lifelong Health and Ageing at University College London, United Kingdom
[2]Population, Policy and Practice, UCL Great Ormond Street Institute of Child Health, London WC1N 1EH, UK
[3]Centre for Population Health Research, Sansom Institute, University of South Australia, Adelaide, Australia
[4]Wolfson Institute of Preventive Medicine, Queen Mary University of London, United Kingdom
[5]University of Exeter Medical School, Exeter, United Kingdom
[6]Institute for Social and Economic Research (ISER), University of Essex, Colchester, United Kingdom
[7]Division of Clinical Epidemiology and Aging Research, German Cancer Research Center (DKFZ), Heidelberg, Germany
[8]Network Aging Research (NAR), University of Heidelberg, Heidelberg, Germany
[9]Institute of Health Care and Social Sciences, FOM University, Essen, Germany
[10]Department of Epidemiology and Public Health, University College London, United Kingdom
[11]Division of Internal Medicine, University Hospital of North Norway, Tromsø, Norway
[12]Endocrinology Research Group, Department of Clinical Medicine, Faculty of Health Sciences, UiT The Arctic University of Norway, Tromsø, Norway
[13]Center for Life Course Health Research, University of Oulu, Oulu, Finland
[14]Oulu University Hospital, Oulu, Finland
[15]Department of Medical Epidemiology and Biostatistics, Karolinska Institutet, Stockholm, Sweden

[16]Department of Clinical Chemistry, Fimlab Laboratories and Faculty of Medicine and Life Sciences, University of Tampere, Tampere, Finland
[17]Research Centre of Applied and Preventive Cardiovascular Medicine, University of Turku, Turku, Finland
[18]Department of Surgical Sciences, Uppsala University, Sweden
[19]Department of Psychology and Logopedics, University of Helsinki, Helsinki, Finland
[20]Helsinki Collegium for Advanced Studies, University of Helsinki, Helsinki
[21]Department of Medicine, Internal Medicine, Lausanne University Hospital, Lausanne, Switzerland
[22]Department of Public Health and Nursing, Faculty of Medicine and Health Sciences, Norwegian University of Science and Technology (NTNU), Trondheim, Norway
Sciences, UiT The Arctic University of Norway, Tromsø, Norway
[23]Department of Clinical Medicine, Faculty of Health Sciences, UiT The Arctic University of Norway, Tromsø, Norway
[24]Department of Cardiology, Division of Cardiothoracic and Respiratory Medicine, University Hospital North Norway, Tromsø, Norway
[25]INSERM, U1018, Centre for Research in Epidemiology and Population Health, Hôpital Paul Brousse, France
[26]Department of Pediatrics, Tampere University Hospital and Faculty of Medicine and Life Sciences, University of Tampere, Tampere, Finland
[27]Department of Clinical Physiology, Tampere University Hospital and Faculty of Medicine and Life Sciences, University of Tampere, Tampere, Finland
[28]Department of Public Health and Caring Sciences, Uppsala University, Sweden
[29]Department of Medical Sciences, Uppsala University, Sweden
[30]Department of Medicine, Division of Cardiovascular Medicine, Stanford University School of Medicine, Stanford, CA 94305, USA
[31]Unit of Population Epidemiology, Division of Primary Care Medicine, Department of Community Medicine, Primary Care and Emergency Medicine, Geneva University Hospitals, Geneva
[32]Department of Epidemiology, Rollins School of Public Health, Emory University, Atlanta, GA 30322, USA
[33]Department of Ambulatory Care and Community Medicine, University of Lausanne, Lausanne, Switzerland
[34]Division of General Neurology, Department of Neurology, General Hospital and Medical University of Graz ,8036, Austria
[35]Research Unit for Genetic Epidemiology, Institute of Molecular Biology and Biochemistry, Center of Molecular Medicine, Medical University of Graz, 8010 Graz, Austria
[36]Department of Neurology, Clinical Division of Neurogeriatrics, Medical University Graz, Austria
[37]Department of General Practice and Primary Health Care, University of Helsinki and Helsinki University Hospital, 00014 University of Helsinki, Finland
[38]Folkhälsan Research Center, Helsinki, Finland
[39]Department of Clinical Physiology and Nuclear Medicine, University of Turku, Turku, Finland
[40]Research Unit of Biomedicine, University of Oulu, Oulu, Finland
[41]Biocenter Oulu, University of Oulu, Oulu, Finland
[42]Medical Research Center (MRC) and Oulu University Hospital, Oulu, Finland
[43]Department of Gastroenterology and Metabolism, Poznan University of Medical Sciences, Poznan, Poland

[44]Department of Epidemiology and Biostatistics, MRC–PHE Centre for Environment & Health, School of Public Health, Imperial College London, London, UK
[45]Research Unit of Clinical Neuroscience, Department of Psychiatry, University of Oulu, Oulu, Finland
[46]Department of Psychiatry, University Hospital of Oulu, Oulu, Finland
[47]Clinicum, University of Helsinki, FI-00014, Finland
[48]South Australian Health and Medical Research Institute, Adelaide, Australia

Corresponding authors:
Jane Maddock PhD: MRC Lifelong Health and Ageing at University College London, United Kingdom. jane.maddocdk@ucl.ac.uk
Elina Hyppönen PhD: Centre for population Health Research, Sansom Institute, University of South Australia, Adelaide, Australia. Elina.Hypponen@unisa.edu.au

Contents

1 Description of cohorts with 25-hydroxyvitamin D data 5
1.1. 1958 British birth cohort (1958BC) 5
1.2. The Colaus Study (COLAUS) 6
1.3. ESTHER 6
1.4. Helsinki Birth Cohort Study (HBCS) 8
1.5. Northern Finland birth cohort 1966 9
1.6. The Prospective Investigation of the Vasculature in Uppsala Seniors (PIVUS) Participants 9
1.7. The Tromsø Study (Tromsø) 10
1.8. Uppsala Longitudinal Study of Adult Men (ULSAM) 11
1.9. Young Finns Study (YFS) 12
2 Description of cohorts without 25-hydroxyvitamin D data 13
2.1. Austria Stroke Prevention Study (ASPS) 13
2.2. English Longitudinal Study of Ageing (ELSA) 14
2.3. Health and Retirement Study (HRS) 15
2.4. Swedish Twin Registry (STR) 16
2.5. TwinGene 17
2.6. UK Biobank 18
2.7. The UK Household Longitudinal Study (Understanding Society) UKHLS 19
2.8. Whitehall II (WII) 20
Supplementary Table 1: Genetic variants quality checks 21
Supplementary Table 2: 25(OH)D, sex specific tertiles 23
Supplementary Table 3. Association between 25(OH)D and cognitive function 24
Supplementary Table 4. Metagression 25
Supplementary Table 5. Association between genetic variants and cognitive function 26
Supplementary Table 6: Association between genetic variants and 25(OH)D 29
Supplementary Table 7. Power calculation 30
Supplementary figure 1. Stratified association of CYP2R1, DHCR7 and synthesis score with global cognition (A) and memory cognition (B) 32
Supplementary figure 2. Vitamin D SNPs and cognitive domain-specific effects in UK Biobank 33
Additional funding sources 34
Study-specific acknowledgments 34
Personal funding statements 34

1 Description of cohorts with 25-hydroxyvitamin D data

1.1. 1958 British birth cohort (1958BC)

Participants

The 1958BC aimed to include all births during one week in March in England, Scotland and Wales (~17,000).[1] Participants in the study have been followed regularly with information collected on a wide-range of factors related to health, lifestyle, growth and development. At 45 years (August 2002 to March 2004) cohort members were invited to a biomedical survey during which time blood samples were collected. Cognitive tests were carried out when participants were 50 years.

25-hydroxyvitamin D

Serum 25(OH)D concentrations were measured using automated application of the IDS OCTEIA ELISA on the Dade-Behring BEP2000 analyzer (sensitivity of 5.0nmol/L, linearity ≤155nmol/L, and intra-assay CV 5.5-7.2%), with adjustment according to the mean of the Vitamin D External Quality Assessment Scheme.

Outcome

Memory-related cognitive function

- *Immediate word recall* [2]: Recall as many words as possible from a list of 10 common words read out by interviewer
- *Delayed word recall* [2]: Recall as many words as possible from a list of 10 common words read out by interviewer, following a short delay of approximately 5 minutes

Additional tests for global cognitive function

- *Verbal fluency, animal naming* :[3] Measures how many words related to a category (in this case, types of animals) a participant can produce
- *Letter cancellation* [4]: Measures speed of processing. Participants were instructed to cross out as many target letters (P and W, 65 in total) as possible in one minute

Genotyping

Genotyping participants in the 1958BC came from two main resources; The Wellcome Trust Case Control Consortium (WTCCC) and the Type 1 Genetics Consortium (T1DGC). Details on how genotyping was conducted have been previously published. [5-9] Briefly, in the WTCCC, samples were genotyped with the GeneChip 500K Affymetrix chip using the CHIAMO calling algorithm and T1DGC used the Illumina 550k platform. Samples were excluded if the SNP call rate was <97%, heterozygosity >30%, external discordance with genotype or phenotype data, individuals identified as having a recent non-European ancestry, duplicate samples, identity by state (IBS) >86% i.e. likely relatives, minor allele frequency (MAF) <1%, Hardy-Weinberg p-value $<10^{-7}$.

Covariates

Educational attainment of cohort members was based on the highest qualification obtained by 42 years, or by 33 years if data was missing. Educational attainment was categorised as: none, < O-level or equivalent, O-level or equivalent, A-level or equivalent and > A-level. Depressive symptoms were assessed using the Clinical

Interview Schedule-Revised at 45 years. The CIS-R is a standardised semi-structured questionnaire, assessing symptoms in the previous week, a score of ≥2 indicated presence of depressive symptoms [10, 11].

1.2. The Colaus Study (COLAUS)

Participants

The CoLaus study is a prospective study assessing the prevalence and determinants of cardiovascular risk factors and diseases in the adult population in Lausanne, Switzerland [12]. The baseline study in 2003-2006 included 6,733 representative participants of the population aged 35-75 years. Blood samples were obtained during the baseline study and cognitive tests were conducted on participants aged over 65 years.

25-hydroxyvitamin D

An ultra-high pressure liquid chromatography-tandem mass spectrometry system was developed and validated for the quantification of vitamin D metabolites 25(OH)D3 and 3-epi-25(OH)D3 in human serum samples. [13] The calibrators, 3Plus1 Multilevel Serum Calibrator Set 25-OH-Vitamin D3/D2 (ChromoSystems, Germany), were standardized against the NIST 972 reference material. The inter-day CV% was 4.6% at 40 nmol/L. Analysis for this study was performed on Vitamin D3 levels excluding the epimer 3-epi-25(OH)D3 values.

Outcome

Global cognitive function

- *Mini Mental State Examination (MMSE):*[14] 30-point questionnaire used to screen for cognitive performance

Genotyping

Samples were genotyped on the Affymetrix GeneChip® Human Mapping 500K using the genotype calling algorithm BRLMM. SNPs with call rates of <70% and individuals with call rates <90% were excluded from further analysis. For imputation, only autosomal SNPs that were present in HapMap release 21 were used. The dataset used for imputation was 390,631 genotyped SNPs with call rate>0.9, Hardy–Weinberg P-value>10-7 and MAF>1%. Imputation was performed using IMPUTE version 0.2.0 and CEU haplotypes from HapMap release 21. Analysis for this study adjusted for four principal components.

Covariates

Educational attainment of the cohort members was categorised as: low (basic or apprenticeship); medium (secondary school); or high (university). Although CoLaus had info on depression,[15] due to a significant drop in sample size, it was not used in this study.

1.3. ESTHER

Epidemiologic study assessing chances of prevention, early detection, and treatment of chronic diseases among older adults (Epidemiologische Studie zu Chancen der Verhütung, Früherkennung und optimierten Therapie chronischer Erkrankungen in der älteren Bevölkerung ,ESTHER)

Participants

ESTHER is a population-based cohort study conducted in Saarland, Germany. [16] The main aim of ESTHER is to contribute information for the prevention, early detection and treatment of chronic diseases.[17, 18] Participants were recruited during health screening visits to their general practitioners. The baseline sample (2000-2002) included 9,949 participants aged between 50 and 74 years, in which blood samples were collected. Cognitive tests on participants aged ≥65 years were conducted during the follow-up study (2005-2008).

25-hydroxyvitamin D

Serum 25(O)D concentrations were measured with the DiaSorin-Liason (Diasorin, Inc., Stillwater, USA) and the IDS-iSYS (Immunodiagnostic Systems GmbH, Frankfurt Main, Germany) immunoassay for women and men, respectively. Values for 25(OH)D were standardised with liquid chromatography tandem-mass spectrometry (LC-MS/MS) in the Department of Clinical Chemistry, Canisius Wilhelma Hospital, Nijmegen, The Netherlands. [19] In ESTHER, 25(OH)D concentrations of 780 women with 0 to 29.5nmol/l were recorded as 29.54nmol/l due to the lower detection limit of the Diasorin assay employed to measure the serum sample in 2001. The assay used for men did not have this detection limit. Additionally, in ESTHER, those with >250nmol/l (*n*=6) were excluded from analyses.

Outcome

The Cognitive Telephone Screening Instrument (COGTEL) [16] was used to assess cognitive function. It consists of the following parameters:

Memory-related cognitive function

- *Verbal short-term memory:*[20] Recall from a list of 8 word pairs following a short delay
- *Working memory* :[21] In reverse order, recall sequences of single-digit numbers which are presented at a 1 second rate
- *Verbal long-term memory:* [20] Recall from a list of 8 word pairs following a delay during which the other tests were conducted

Additional tests for global cognitive-function

- *Verbal fluency* :[22] Includes two tests, letter fluency i.e. producing as many words as possible beginning with a given letter within 60 seconds and category fluency i.e. producing as many different types of professions as possible within 70 seconds
- *Inductive reasoning:* [21] Involves the continuation of a mathematical sequence

Genotyping

Samples were genotyped using the TaqMan OpenArrayTM SNP Genotyping Platform (Applied Biosystems, Foster City, CA) according to the manufacturer's instructions. The overall call rate was 97.3%. The genotyping concordance was 100%.

Covariates

Educational attainment was categorised as ≤9, 10-11 or ≥12 years in education at mean age of 62.1(SD6.6). Depressive symptoms were assessed using 15-item Geriatric Depression Scale. This is specifically designed for rating depression in the elderly. Cut-off score of 5 was used.[23]

1.4. Helsinki Birth Cohort Study (HBCS)

Participants

The HBCS is a longitudinal study focused on the early origins of health and disease.[24] Information for this study was collected using data from the maternity hospital, clinical, school databases and other registries on people born in 1934-1944 in two hospitals in Helsinki Finland. Blood samples and cognitive testing were administered during the clinical follow-up study (2001-2003) of 2,003 men and women. Analysis for this study excluded those with a history of stroke (*n*=107) and those with a discrepancy between reported gender and gender determined by their genotype (*n*=8).

25-hydroxyvitamin D

Serum 25(OH)D concentrations were measured using the Chemiluminescent Microparticle Immuno Assay (CMIA, Abbott Laboratories, Abbott Park, IL, USA). Analyses were done with an Architect ci8200 –analyzer. The intra-assay CV is 4.3 % + 1.6 % and the detection limit was 5.5 nmol/l.

Outcome

The HBCS uses CERAD (The Consortium to Establish a Registry for Alzheimer's disease) which consists of the following tests:

Memory-related cognitive function

- *Immediate verbal recall*: Recall as many words as possible from list of 10 words in 90 seconds.
- *Delayed verbal recall*: Recall as many words as possible from list of 10 words after a delay
- *Verbal Learning*: Participant is shown a list of 10 words 3 times then recalls as many words as possible. Total score is the number of correct words recalled.

Additional tests for global cognitive function

- *Verbal fluency, animal naming*: Measures how many words related to a category (in this case, types of animals) a participant can produce

Genotyping

Genotyping was performed with a modified Illumina 610k chip by the Wellcome Trust Sanger Institute, Cambridge, UK. Genetically related individuals and those with gender discrepancies were excluded. After quality control procedures 1,720 samples remained for the analyses. SNPs were excluded if they met the following conditions: call rate < 0.95, minor allele frequency < 0.01, and HWE test with P < 1x10-5. Genomic coverage was extended to ~2.5M common SNPs by imputation using the HapMap phase II CEU data (NCBI build 36 (UCSC hg18)) as the reference sample and MACH software. SNPs with low imputation quality (r-squared < 0.30), low minor allele frequency (MAF < .01), or that diverged from HWE (1x10-5), were excluded from the analyses. Analyses were adjusted for principal components.

Covariates

Educational attainment was categorised as: folk school/elementary/middle school; learning profession; elementary school or similar; or lower; or higher university degree. Depressive symptoms were assessed using Centre for Epidemiologic Studies Depression Scale with a cut-off of <16.

1.5. Northern Finland birth cohort 1966

Participants

The NFBC66[1] is one of the Northern Finland cohorts forming a longitudinal research program aiming to promote health and well-being in the population. The original cohort consisted of 12,231 people born in 1966, with blood samples available for 6,000 participants at 31 years.[25] Cognitive testing was conducted during the 46-year follow-up.

25-hydroxyvitamin D

25(OH)D2 and 25(OH)D3 were measured in four batches using high-performance liquid chromatography-tandem mass spectrometry. Further details have been previously published.[26] Analyses for this study were adjusted for vitamin D batch.

Outcome

- *Paired Associates Learning (PAL):* assess visual memory and new learning

Genotyping

Genotyping was conducted using Infinium 370cnvDuo, keeping MAF > 0.01, call rate > 0.95 (0.99 for MAF < 0.05), HWE p-value > 1e-6. Subjects with overall call rate < 0.95 and gender mismatch were excluded.

Covariates

Educational attainment was categorised into: non occupational education or vocational training course; vocational school or post-secondary education; polytechnic education or university degree. Presence of depression (yes, no) was assessed from responses to following question: "*Have you ever had any of the following symptoms, sicknesses or injuries verified or treated by a doctor: depression?"*

1.6. The Prospective Investigation of the Vasculature in Uppsala Seniors (PIVUS) Participants

The PIVUS[2] study includes men and women aged 70 years in Uppsala, Sweden. The primary aim of this study was to evaluate the usefulness of different measurements of endothelial function and other techniques to evaluate vascular function. The baseline study (2001-2004) consisted of 1,016 participants in which blood samples were obtained. Cognitive tests were conducted in the follow-up sweep (~75ys).

25-hydroxyvitamin D

Serum 25(OH)D was measured at the department of Clinical Chemistry at Uppsala University Hospital using the DiaSorin-Liason immunoassay. CV for inter-assay analyses is 18.4% at a 25(OH)D level of 39.5 nmol/L and 11.7% at 121.2 nmol/L. The intra-assay CV is 7.1% at 44.7 nmol/L and 3.6% at 120.0 nmol/L.

Outcome

Memory-related cognitive function

[1] http://www.oulu.fi/nfbc/
[2] http://www.medsci.uu.se/pivus/

- *Relevant sub-tests of 7 minute Screen test*:[27] enhanced cued recall i.e. identification and recall of 16 pictures immediately and after an interval with semantic cues if necessary

Additional tests for global cognitive function

- *Mini Mental State Examination (MMSE):*[14] 30-point questionnaire used to screen for cognitive impairment
- *Relevant sub-tests 7 minute Screen test* :[27] Consists of (1) Benton temporal orientation i.e. measurement of orientation in time (2) Clock drawing i.e. subject draws the face of a clock and places the hands on a fixed time (3) Verbal fluency i.e. participant names as many different animals as possible in one minute
- *TMT A*: Participant is asked to draw lines with a pencil between the numbers in the right order as fast as possible. The score is equal to the time in seconds. TMT A consists of digits 1-25
- *TMT B*: Participant is asked to draw lines with a pencil between the numbers in the right order as fast as possible. The score is equal to the time in seconds. TMT B consists of digits and letters 1-A-2-B etc.

Genotyping

SNPs were genotyped as part of a larger study at the SNP technology platform at Uppsala University (http://www.genotyping.se/) on an Illumina BeadStation 500GX using Infinium iSelect and Golden Gate assays from Illumina Inc. [28] Genotyping calls were performed with Illumina BeadStudio or GenCall software. Samples with low call rate (<90%), excess heterozygosity or cryptic relatedness were excluded from analyses. SNPs with a call rate less than 90%, or that failed HWE (exact p-value<1x10-6) were excluded from the study.

Covariates

Educational attainment was categorised as primary school (<9 years), secondary school (9-12 years), university level (>12 years) at mean age of 70.2(SD0.2). There was no information on depressive symptoms.

1.7. The Tromsø Study (Tromsø)

Participants

Tromsø is a longitudinal population-based multi-purpose study focusing on lifestyle-related diseases in Norway. It began in 1974 with 6,595 male participants. Blood collection was conducted in Tromsø 4 (1994-1995, *n*=27,158)[29] and cognitive tests were conducted in Tromsø 5 (2001, *n*=8,130).[29]

25-hydroxyvitamin D

Serum 25(OH)D concentrations were measured by an electrochemiluminescence immunoassay (ECLIA), using an automated clinical chemistry analyser (Modular E170, Roche Diagnostics®, Mannheim, Germany). The total analytical CV for the 25(OH)D assay was 7.3 %. In Tromsø, 25(OH)D concentrations have been shown to be overestimated in smokers, therefore models with 25(OH)D analysed in Tromsø were controlled for smoking status.[30]

Outcome

Memory-related cognitive function

- *12 word memory test*:[31] Words were shown written on a board and pronounced. Participants had 2 minutes to recall the word

Additional tests for global cognitive function

- *Finger tapping test:*[31] Participant is asked to tap as many times as possible in 10 seconds with their index finger on a computer
- *Digit symbol coding test:*[21] Consists of rows containing blank squares, each paired with a randomly assigned number from one to nine, and a printed key about that pair, each number with a different nonsense symbol. Participants consecutively fill-in as many as possible of the blank spaces with the corresponding symbol as quickly as possible for 90 seconds

Genotyping

Genotyping was conducted by KBioscience (http://www.kbioscience.co.uk) using KASP (KBioScience Allele-Specific Polymorphism) SNP genotyping system. Two separate manual quality control checks were performed, and the data was also checked by specific software to determine that there were no incorrect call assignments, no samples too close or too far from the origin, no NTCs amplified, or any incorrect calls. The call rate for all the 4 SNPs was >98 % and the Hardy-Weinberg equilibrium p value was p> 0.01.

Covariates

Educational attainment was a continuous variable measuring years of education by mean age of 64.5(SD9.9). Depressive symptoms were assessed using Hopkins symptom checklist-10 which is a self-reported symptom inventory consisting of symptoms commonly observed in the population[32] with responses as yes or no.

1.8. Uppsala Longitudinal Study of Adult Men (ULSAM)

Participants

ULSAM is a longitudinal study based on all men born between 1920 and 1924 in Uppsala, Sweden. The study began in 1970 when the men were 50 years (*n*=2,322).[33] The main aim of the study was to identify cardiovascular risk factors. Blood collection and cognitive testing was conducted when participants were age 70 years (1991-1995).

25-hydroxyvitamin D

Serum 25(OH)D concentrations were determined with high-pressure liquid chromatography (HPLC) atmospheric pressure chemical ionization (APCI) mass spectrometry (MS) at Vitas, Oslo, Norway (www.vitas.no). HPLC was performed with a Hewlett Packard 1100 liquid chromatography (Agilent Technologies, Palo Alto CA, USA) interfaced by APCI to a Hewlett Packard mass spectrometer operated in single-ion monitoring mode (SIM). Recovery is 95%; the method is linear from 5-400 nmol/L and the limit of detection is 1-4 nmol/L. The CVs for inter-assay analyses were 7.6% at 25(OH)D concentrations of 47.8 nmol/L and 6.9% at 83.0 nmol/L. The intra-assay CV was 5.1% at 47.8 nmol/L and 6.1% at 83.0 nmol/L. The assay is accredited by DEQAS.

Outcome

Global cognitive function

- *MMSE:* [14] 30-point questionnaire used to screen for cognitive impairment
- *TMT A:* [34] Participant is asked to draw lines with a pencil between the numbers in the right order as fast as possible. The score is equal to the time in seconds. TMT A consists of digits 1-25
- *TMT B:* [34] Participants is asked to draw lines with a pencil between the numbers in the right order as fast as possible. The score is equal to the time in seconds. TMT B consists of digits and letters 1-A-2-B etc.

Genotyping

As for PIVUS, SNPs were genotyped at the SNP technology platform at Uppsala University (http://www.genotyping.se/) on an Illumina BeadStation 500GX using Infinium iSelect and Golden Gate assays from Illumina Inc. Genotyping calls were performed with Illumina BeadStudio or GenCall software. Samples with low call rate (<90 %), excess heterozygosity or cryptic relatedness were excluded from analyses. SNPs with a call rate less than 90%, or that failed (exact P-value<1x10-6) were excluded from the study.

Covariates

Educational attainment was categorised as 7, 8-10 or ≥12 years in education. Depressive symptoms were assessed using International classification of disease[35] with responses as yes or no.

1.9. Young Finns Study (YFS)

Participants

The Cardiovascular Risk in Young Finns Study (YFS) is an ongoing longitudinal population-based study focusing on cardiovascular risk factors from childhood to adulthood. The study was originally designed as a national collaborative effort between all university hospitals and several other institutions in Finland. The first cross-sectional study of the YFS was performed in 1980, and it included 3,596 randomly selected children and adolescents (both boys and girls) aged 3, 6, 9, 12, 15 and 18 years. Until the year 2011, the cohort has been regularly followed-up in 3-9 year intervals. More detailed information on the YFS study population and protocol is reported elsewhere.[36] Blood samples were collected in the 2007 wave and cognitive tests were conducted in 2011.

25-hydroxyvitamin D

Blood samples were stored at 70°C and analysed in 2008. Serum 25(OH)D was analysed by radioimmunoassay (RIA) (DiaSorin, Inc.). The limit of detection was 3.8 nmol/L. The interassay coefficient of variation (CV) was 8.5% (n=128) at the level of 35.7 nmol/L, and 8.8% (n =113) at the level of 135.3 nmol/L.[37]

Outcome

Memory-related cognitive test

- *Paired associates learning (PAL) test*: assessed visual and episodic memory as well as visuospatial associative learning containing aspects of both delayed response procedure and conditional learning. During the PAL test, either 1, 2, 3, 6, or 8 patterns were displayed sequentially in boxes placed on the screen. After that, the patterns were presented in the center of the screen, and the participants were supposed to point the box in which the particular pattern was previously seen. The test moves on to the next stage if all the patterns were placed to right boxes. In case of incorrect response, all the patterns were re-displayed in their original locations and another recall phase was followed. The test terminated if the patterns were still incorrectly placed after 10 presentation and recall phases

Additional tests for global cognitive function

- *Spatial working memory (SWM) test:* measures ability to retain spatial information and to manipulate items stored in the working memory, problem solving as well as the ability to conduct a self-organized search strategy. During this test the participants were presented with randomly distributed coloured boxes ranging in number from 4 to 8. After that, the participants searched for tokens hidden in the boxes. When a token was found it was to be moved to fill an empty panel on the right-hand side of the screen. Once the token had been moved from the box, the participant had to recall that the computer would never hide a new token in a box that previously contained one; therefore the participants were not supposed to revisit the same boxes again

- *Reaction time (RTI) test:* assesses speed of response and movement on tasks where the stimulus was either predictable (simple location task) or unpredictable (five-choice location task). In the first part of this test, a large circle was presented in the center of the screen. The participant was supposed to press a button on a press pad until a small yellow spot appears in the large circle. When the yellow spot appeared the participant was supposed to touch the spot as soon as possible with the same hand that was pressing the button on the press pad. In the second part of the test, the same task was performed, except that in this part five large circles were presented on the screen, and the small yellow spot might appear in any of the five circles. Again the participant was supposed to touch, as soon as possible, the yellow spot with the hand pressing the button on the press pad
- *Rapid visual information (RVP) test*: assess visual processing, recognition and sustained attention. In this test the participant was presented with a number sequence (*e.g.* 3, 5, 7) next to a large box where numbers appeared in a random order. Whenever the particular sequence was presented, the participant was supposed to press a button on a press pad. At the beginning, the participant was given visual cues (*i.e.* colored or underlined numbers) to help to recognize the particular sequence. When the test proceeded, the cues were removed.

Genotyping

Genomic DNA was extracted from peripheral blood leukocytes using a commercially available kit and the Qiagen BioRobot M48 Workstation according to the manufacturer's instructions (Qiagen, Hilden, Germany). Genotyping was performed on 2,556 samples using a custom-built Illumina Human 670k BeadChip at the Welcome Trust Sanger Institute. Genotypes were called using the Illuminus clustering algorithm. Fifty-six samples failed to meet the Sanger genotyping pipeline QC criteria (i.e. duplicated samples, heterozygosity, low call rate, or Sequenom fingerprint discrepancy). Out of the remaining 2,500 samples, one failed the gender check, and three were removed due to low genotyping call rate (< 0.95) and 54 for possible relatedness (pi-hat $>$ 0.2). Based on the Hardy-Weinberg equilibrium (HWE) test, 11,766 SNPs were excluded ($p < 1e-6$), and 7,746 SNPs failed the missingness test (call rate < 0.95) and another 34,596 SNPs failed the frequency test (MAF $<$ 0.01). After quality control, 2,442 samples and 546,677 genotyped SNPs were available for further analysis. Genotype imputation was performed using IMPUTE2 and 1000 Genomes Phase I Integrated Release Version 3 (March 2012) haplotypes as a reference. [38] Analyses adjusted for principle components.

Covariates

Educational attainment was categorised as: low level (vocational school, vocational college); medium level (university or applied sciences, university studies (no final degree), Bachelors degree); or high level(Masters, Licentiate degree, Doctoral degree), Depression data were taken from self-reported data on depression diagnoses in 2007.

2 Description of cohorts without 25-hydroxyvitamin D data

2.1. Austria Stroke Prevention Study (ASPS)

Participants

The ASPS is a prospective study on the effects of vascular risk factors on brain structure and function in cognitively normal inhabitants of Graz, Austria. [39, 40] 2,007 participants aged 45 to 86 years were recruited from the official community register and all participants were free of stroke and dementia. Individuals were excluded from the study if they had a history of neuropsychiatric disease. Between 1991 and 1994, 509 participants randomly selected from the entire cohort underwent cognitive testing. To enlarge the cohort with imaging and neuropsychological assessments, an additional 567 individuals were randomly selected in a second study

between 1999 and 2003. Participants of the first and second panels were pooled, which resulted in a total of 1,076 individuals with blood and cognitive tests.

Outcome

Memory-related cognitive function

- *Word association*: Assessed during Bäumler's Lern-und Gedächtnistest (LGT-3) [40] to measure verbal memory
- *Digit association*: Assessed during LGT-3 to measure verbal memory
- *Story recall*: Assessed during LGT-3 to measure verbal memory
- *Trail recall*: Assessed during LGT-3 to measure visuospatial memory
- *Design recall*: Assessed during LGT-3 to measure visuospatial memory

Additional tests for global cognitive function

- *Wisconsin Card Sorting test*: Used as a measure of conceptional reasoning.
- *The Alters–Konzentrations–Test*: Used to test attention and speed
- *Trail Making Test B (TMT B):* Participants are asked to draw lines with a pencil between the numbers in the right order as fast as possible. The score is equal to the time in seconds. TMT B consists of digits and letters 1-A-2-B etc.
- *Digit span*: Used to test attention and speed
- *Complex reaction time*: Participants react selectively to a specific combination of a visual and acoustic signal by pressing a button as quickly as possible

Genotyping

Genotyping was done at the Human Genotyping Facility, Genetic Laboratory Department of Internal Medicine, Erasmus MC, Rotterdam, The Netherlands, using the Illumina Human610-Quad BeadChip. Participant-specific quality controls included filters for call rate (<98%), and high autosomal heterozygosity (FDR <1%). Quality filters used a call rate >98%, a minor allele frequency >0.01 and a Hardy-Weinberg p>1x10-6. In total, 550635 markers and 856 people passed all criteria. ASPS used the Markov Chain Haplotyping (MaCH) package(http://www.sph.umich.edu/csg/abecasis/MACH, version 1.0.15 for imputation to plus strand of NCBI build 36, HapMap release #22.

Covariates

Educational attainment was categorised as 9, 10, 13 or 18 years in education. Depressive symptoms were assessed using Eigenschaftswörterliste (range 0-15). This is a validated multidimensional tool consisting of a list of given adjectives describing the emotional status of a test person at the time of the interview.[41]

2.2. English Longitudinal Study of Ageing (ELSA)

Participants

ELSA is an English based study consisting of men and women aged 50 years and over. It is a follow-up study of respondents to the Government's Health Survey for England (HSE)[3], an annual cross-sectional survey designed to be representative of the community-based population in England. The main objective of ELSA is to collect longitudinal multidisciplinary data relating to health, disability, biological markers, well-being, social participation and economics. ELSA began in 2002 and has been followed up every two years. The sample consists of individuals seen in HSE in 1998, 1999 or 2001. 11,391 participants were interviewed for wave 1

[3] http://www.dh.gov.uk/

(2002) in which cognitive testing was conducted.[42] 8,780 participants were interviewed in wave 2 (2004), in which blood samples from 7966 participants were taken for DNA analysis.[42]

Outcome

Memory-related cognitive function

- *Immediate word recall:*[2] Recall as many words as possible from a list of 10 common words read out by interviewer
- *Delayed word recall:*[2] Recall as many words as possible from a list of 10 common words read out by interviewer, following a short delay of approximately 5 minutes

Additional tests for global cognitive function

- *Verbal fluency, animal naming*:[3] Measures how many words related to a category (in this case, types of animals) a participant can produce
- *Letter cancellation:* [4] Measures speed of processing. Participants were instructed to cross out as many target letters (P and W, 65 in total) as possible in one minute.

Genotyping

DNA was extracted from blood samples using magnetic bead technology (Medical Solutions, Nottingham). Genotyping was performed using the KASPar methodology. The call rates and the concordance rates of the four SNPs were >98%, HWE values were $p>0.08$.

Covariates

There was no information on educational attainment. Depressive symptoms were assessed using the 8-item Centre for Epidemiologic Studies Depression Scale. This is a short self-report scale design to measure depressive symptomatology based on the past week in the general population with higher summary scores indicating greater symptoms.[43] A cut-off score of 3 was used and has been shown to be clinically significant for 8-item questionnaire and cut-off of 16 for longer version.[44]

2.3. Health and Retirement Study (HRS)

Participants

The HRS is an ongoing nationally representative study with biannual assessments as previously described.[45] Data for this study comes from older Americans with European ancestry who participated in the 2006 wave of the US HRS.[4]

Outcome

Memory-related cognitive tests[5]

- *Immediate word recall:* Recall as many words as possible from a list of 20 common words read out by interviewer

---

[4] http://hrsonline.isr.umich.edu/index.php?p=start
[5] http://hrsonline.isr.umich.edu/modules/meta/xyear/cogimp/desc/COGIMPdd.pdf

- *Delayed word recall:* Recall as many words as possible from a list of 20 common words read out by interviewer, following a short delay of approximately 5 minutes

Additional tests for global cognitive function[6]

- *Date naming:* name month, day, year and day of week
- *Object naming*
- *Naming of the current President and Vice President of the United States*
- *Serial 7's test:* The interviewer asked the respondent to subtract 7 from 100, and continue subtracting 7 from each subsequent number for a total of five trials. It was up to the respondent to remember the value from the prior subtraction, such that the interviewer did not repeat the difference said by the respondent after each trial.
- *Backwards count:* respondents were asked to count backwards for 10 continuous numbers beginning with the number 20.

Genotyping

DNA samples from buccal swabs were genotyped in batches at the National Institutes of Health Center for Inherited Disease Research using the Ilumina Human Omni-2.5 Quad beadchip.[7] Analyses were adjusted for principle components

Covariates

Educational attainment was categorised into number of tertiles based on number of years in school. Depression was based on CESD (0-8) total score with a cut-off of 4 or more indicative of depression if <2 of the 8 items missing as previously described .[46]

2.4. Swedish Twin Registry (STR)

Participants

The participants of STR were taken from longitudinal twin studies of aging. All these studies have previously been described in detail and are sub-samples of the population based Swedish Twin Registry.[47-49] The study consists of The Swedish Adoption/Twin Study of Aging (SATSA) with up to ten longitudinal occasions and the GENDER study with three interview occasions. All participants are of European ancestry and born in Sweden. For these sub-samples, individuals participated in at least one in-person session and in which a blood sample was drawn. Analyses were adjusted for study name i.e. SATSA or GENDER. Blood samples and cognitive tests were taken at wave 1 in GENDER and at the first availability from wave 3 to wave 9 in SATSA.

Outcome

Memory-related cognitive function

- *Immediate word recall*
- *Delayed word recall*
- *Thurstone's memory test:* Subjects are shown 28 pictures and then asked for recognition of these among others. The pictures were enlarged from the original version to minimize any possible visual problems

[6] http://hrsonline.isr.umich.edu/modules/meta/xyear/cogimp/desc/COGIMPdd.pdf
[7] http://hrsonline.isr.umich.edu/index.php?p=xxgen1

Additional tests for global cognitive function

- General cognitive ability was calculated from principal component analysis of four tests (Synonyms, Block Design, Thurstone Picture Memory and Symbol Digit in GENDER, and from constructing the first principal component of nine cognitive tests in SATSA (Analogies, Synonyms, Information, Block Design, Card Rotations, Digits Span (Forward and Backward), Thurstone Picture Memory, Symbol Digit, and Figure Identification).

Genotyping

Samples were genotyped using Metabochip, which were used in Study II. [50] Genotyping was performed at Uppsala University SNP Technology Platform using the Illumina iSelect Metabochip genotyping array. Call rate > 90%.

Covariates

Educational attainment was categorised as: elementary or middle school; high school or equilivant; college or higher. Presence of depression was examined through review or medical record, CES-D, depression diagnosis in the National Patient Register

2.5. TwinGene

Participants

The TwinGene project, conducted between 2004 and 2008, is a population-based Swedish study of twins born between 1911 and 1958.[51] The study participants have previously participated in a telephone interview called Screening Across the Lifespan Twin Study, conducted between 1998 and 2002. To be included in TwinGene, both twins within a pair had to be alive. The zygosity of the twins was based on self-reported childhood resemblance, or by using DNA markers (for 18% of the total sample). In total, 12,591 individuals participated by donating blood to the study, and by answering questionnaires about life style and health. The study was approved by the local ethics committee at Karolinska Institutet and all participants gave informed consent.

Outcome

Memory-related cognitive function

- *Immediate word recall*
- *Delayed word recall*

Additional tests for global cognitive function

- *Cognitive screen score:*[8] Constructed from immediate word recall, delayed word recall, similarity test (see footnote for full details).

Genotyping

TwinGene participants were genotyped with Illumina Human OmniExpress (≈700,000 SNPs); genotype rate per SNP greater than 95%; genotype rate per individual greater than 97%.

---

[8] https://dornsife.usc.edu/assets/sites/342/docs/Tele.pdf

Covariates

Educational attainment was categorised as: elementary or middle school; high school or equivalent; college or higher. Depression was assessed through CES-D, antidepressant use and doctor screening items.

2.6. UK Biobank

Participants

UK Biobank is a large prospective study with over 500,000 participants from across the United Kingdom aged 40–69 years at recruitment in 2006–2010[9]. The study has data from questionnaires, physical measures, sample assays, accelerometry, multimodal imaging, genome-wide genotyping and longitudinal follow-up for a large number of health-related outcomes. In the current study, we use the interim UK Biobank genetic data that comprise more than 150,000 samples. We have restricted the analyses to individuals genetically defined as Caucasian and to persons that are unrelated.

Outcome

Memory-related cognitive tests

- *Pairs matching tests:*[10] This category contains data on 'pairs' matching tests. Participants are asked to memorise the position of as many matching pairs of cards as possible. The cards are then turned face down on the screen and the participant is asked to touch as many pairs as possible in the fewest tries. Multiple rounds were conducted. The first round used 3 pairs of cards and the second 6 pairs of cards. In the pilot phase an additional (i.e. third) round was conducted using 6 pairs of cards. however this was dropped from the main study as the extra set of results were very similar to the second and not felt to add significant new information. This category includes data on the number of columns (i.e. 3 or 4), number of rows (i.e. 2 or 3) and the number of correct and incorrect matches.

Additional tests for global cognitive function

- *Reaction time:*[11] This category contains data on a test to assess reaction time and is based on 12 rounds of the card-game 'Snap'. The participant is shown two cards at a time; if both cards are the same, they press a button-box that is on the table in front of them as quickly as possible. For each of the 12 rounds, the following data were collected: the pictures shown on the cards (Index of card A, Index of card B), the number of times the participant clicked the 'snap' button, and the time it took to first click the 'snap' button.

Additional cognitive tests for sensitivity analyses

- Fluid intelligence (i.e. Reasoning)[12]: This tests the capacity to solve problems requiring reasoning, logic and acquired knowledge. The participant is given two minutes to complete as many questions as possible
- *Prospective memory:* Information on how the prospective memory test was conducted can be found: http://biobank.ctsu.ox.ac.uk/crystal/label.cgi?id=100031

---

[9] https://www.ukbiobank.ac.uk/
[10] https://biobank.ctsu.ox.ac.uk/crystal/label.cgi?id=100030
[11] https://biobank.ctsu.ox.ac.uk/crystal/label.cgi?id=100032
[12] http://biobank.ctsu.ox.ac.uk/crystal/label.cgi?id=100027

Genotyping

Approximately 450,000 of the participants have been/are being genotyped using the UK Biobank Axiom array from Affymetrix. There are approximately 800,000 markers on this array. The other approximately 50,000 samples were genotyped on the closely related UK BiLEVE array. These are two very similar arrays with more than 95% common marker content. The analysis sample was restricted to unrelated individuals, based on a threshold of 0.05 estimated from genetic kinships, and to individuals of Caucasian genetic ancestry using principal components analyses (PCA). Analyses were adjusted for principle components.

Covariates

Educational attainment categorised as: none, NVQ/CSE/O-levles/A-levels or degree/professional. Presence of depression was obtained from the question "*In the last 2 weeks, how often have you felt down, depressed or hopeless*? With the responses: several, more than half the days, nearly every day or, not at all.

2.7. The UK Household Longitudinal Study (Understanding Society) UKHLS

Participants

The UKHLS is an annual longitudinal survey of over 40,000 UK households, beginning in 2009-2010. Annual interviews collecting sociodemographic information are conducted throughout each year, usually in the same month for each participant. Biomedical measures and blood samples were collected during a single nurse visit five months after the annual wave 2 interview (2010-2012) or wave 3 interview (2011-2013).[52] Cognitive performance tests were carried out during the Wave 3 interview.

Outcome

Memory-related cognitive function

- *Immediate word recall:* Respondent immediately recalls as many words as possible
- *Delayed word recall:* Respondent recalls as many words as possible after a delay

Additional tests for global cognitive function

- *Subtract 7 test:* Participant subtracts 7 from 100, and continue subtracting 7 from each subsequent number for a total of five trials

Genotyping

Genotyping was conducted at the Wellcome Trust Sanger Institute, Hinxton using Illumina Human Core Exome Bead chip. The overall call rate was <98%.

Covariates

Educational attainment was categorised as: Low: no qualification or GCSE and equivalent, Medium: A level and equivalents, High: Undergraduate agree and higher. Depression was categorised as yes or no based on clinical presentation.

2.8. Whitehall II (WII)

Participants

WII was established in 1985 with the aim of investigating the importance of socioeconomic difference in physical, mental illness and mortality in London-based offices of 20 Civil Service departments.[53] DNA preparation and cognitive testing was conducted using information from phase 7 (2002-2004, *n*=6,967).

Outcome

Memory-related cognitive tests:

- *Memory test score*:[54] Recall in writing as many of the 20 two syllable words in any order within two minutes

Additional tests for global cognitive function

- *Alice-Heim 4* :[55] Composed of a series of 65 verbal and mathematical reasoning items of increasing difficulty. It tests inductive reasoning, measuring the ability to identify patterns and infer principles and rules. Participants had 10 minutes to do this section
- *Mill Hill Score:*[56] Vocabulary was assessed using the multiple choice format consisting of a list of 33 stimulus words ordered by increasing difficulty and six response choices
- *Verbal fluency (phonemic):* [57] Recall in writing as many words beginning with 's' as they could in one minute
- *Verbal fluency (semantic):* [57] Recall in writing as many animal names as they could in one minute

Genotyping

DNA was extracted from blood samples using magnetic bead technology (Medical Solutions, Nottingham). Genotyping was performed using the Illumina 50K IBC CVD chip or KASPar methodology. The call rates and the concordance rates of the four SNPs were >98%. The HWE p values were $p>0.07$.

Covariates

Educational attainment was a continuous variable measuring years of education by mean age of 55.5 (SD=5.9) years. Depressive symptoms were assessed using the 30-item General Health Questionnaire [58] with a cut-off of >4.

Supplementary Table 1: Genetic variants quality checks

Study	SNP	Gene	Alleles Measured	MAF	HWE	Imputed/Genotyped
Studies with 25(OH)D:						
1958BC	rs12785878	*DHCR7*	G/T	0·22	0·49	Genotyped
	rs12794714	*CYP2R1*	A/G	0·43	0·59	Genotyped
COLAUS	rs2276360	*DHCR7*	G/C	0·28	0·62	Genotyped
	rs12794714	*CYP2R1*	A/G	0·47	0·73	Imputed
ESTHER	rs11603330	*DHCR7*	A/C	0·26	0·71	Genotyped
	rs12794714	*CYP2R1*	A/G	0·46	0·95	Genotyped
HBCS	rs7944926	*DHCR7*	A/G	0·38	0·43	Genotyped
	rs12794714	*CYP2R1*	G/A	0·39	0·14	Genotyped
NFBC66	rs7944926	*DHCR7*	A/G	0·39	0·03*	Genotyped
	rs11023350	*CYP2R1*	G/T	0·41	0·65	Genotyped
PIVUS	rs12785878	*DHCR7*	C/A	0·35	0·06	Genotyped
	rs12794714	*CYP2R1*	G/A	0·40	0·78	Genotyped
TROMSØ	rs12785878	*DHCR7*	G/T	0·39	0·46	Genotyped
	rs12794714	*CYP2R1*	A/G	0·41	0·34	Genotyped
ULSAM	rs12785878	*DHCR7*	C/A	0·33	0·64	Genotyped
	rs7938266	*CYP2R1*	G/A	0·39	0·62	Genotyped
YFS	rs12785878	*DHCR7*	G/T	0·40	0·17	Genotyped
	rs12794714	*CYP2R1*	A/G	0·38	0·27	Imputed
Studies without 25(OH)D:						
ASPS	rs12785878	*DHCR7*	G/T	0·29	0·19	Imputed
	rs12794714	*CYP2R1*	A/G	0·44	0·72	Imputed
ELSA	rs12785878	*DHCR7*	G/T	0·22	0·08	Genotyped
	rs12794714	*CYP2R1*	A/G	0·43	0·82	Genotyped
HRS	rs12785878	*DHCR7*	G/T	0·27	<0·001*	Imputed
	rs12794714	*CYP2R1*	A/G	0·44	0·62	Genotyped
STR	rs736894	*DHCR7*	T/G	0·30	0·17	Imputed
	rs12794714	*CYP2R1*	A/G	0·39	0·79	Imputed

TwinGene	rs12785878	*DHCR7*	G/T	0·33	0·78	Imputed
	rs12794714	*CYP2R1*	A/G	0·40	0·09	Genotyped
UK Biobank	rs12785878	*DHCR7*	G/T	0·21	0·30	Genotyped
	rs12794714	*CYP2R1*	A/G	0·42	0·97	Genotyped
UKHLS	rs12785878	*DHCR7*	G/T	0·22	0·46	Genotyped
	rs12794714	*CYP2R1*	A/G	0·42	0·43	Genotyped
WII	rs12785878	*DHCR7*	G/T	0·23	0·73	Genotyped
	rs12794714	*CYP2R1*	A/G	0·43	0·49	Genotyped

G: Guanine; T: Thymine; A: Adenine; C: Cytosine; MAF: Minor allele frequency; HWE: Hardy-Weinberg equilibrium

* Sensitivity analysis without NFBC66 and HRS had little impact on the final results

Supplementary Table 2: 25(OH)D, sex specific tertiles

	Sex-specific 25(OH)D tertile 1, nmol/l					Sex -specific 25(OH)D tertile 2, nmol/l					Sex -specific 25(OH)D tertile 2, nmol/l				
**Study**	N	mean	SD	min	max	N	mean	SD	min	max	N	mean	SD	min	max
1958BC	1,881	31·8	8·1	9·5	44·8	1,996	55·3	6·5	43·4	66·5	2,013	86·5	17·3	66·5	187·3
COLAUS	289	24·7	7·3	7·7	36·1	291	47·1	6·9	35·2	60·0	293	76·8	14·7	60·0	147·0
ESTHER	3,117	31·0	5·1	7·0	41·7	3,178	46·5	7·1	35·9	63·6	3,207	74·8	21·9	49·3	225·6
HBCS	340	42·7	7·1	19·0	53·0	345	60·1	4·7	51·0	69·0	350	83·3	18·6	67·0	292·0
NFBC	1,100	34·8	5·5	18·6	43·2	1,103	50·0	3·8	43·2	56·5	1,112	66·9	9·3	56·4	116·2
PIVUS	332	37·4	7·3	10·9	49·7	326	55·9	5·2	45·0	66·0	341	79·1	14·4	61·0	143·0
Tromsø	2,374	38·5	7·8	10·1	50·0	2,390	56·9	4·7	48·2	65·2	2,397	81·1	14·7	65·3	192·2
ULSAM	398	48·6	9·4	5·0	59·9	398	68·1	4·8	60·0	76·4	398	89·4	12·4	76·4	153·3
YFS	651	39·5	7·0	13	49	657	56·3	4·7	48	65	715	79·5	14·4	63	168

Supplementary Table 3. Association between 25(OH)D and cognitive function

Cohort	Cognitive outcome	N	Coefficient, per 10nmo/l decrease in 25(OH)D	(se)	p-value	$p_{curvature}$	$p_{gender\ interaction}$	$p_{age\ interaction}$
**Overall***	Global cognition	15,523	-0·013	(0·009)	0·15	0·001	0·73	0·54
	Memory Cognition	14,081	-0·001	(0·010)	0·89	0·006	0·96	0·65
**1958BC**	Global cognition	4,467	0·010	(0·006)	0·10	0·004	0·49	-
	Memory Cognition	4,557	0·014	(0·006)	0·02	0·01	0·45	-
**CoLaus**	Global cognition	872	0·004	(0·034)	0·02	0·70	0·27	0·60
**ESTHER**	Global cognition	1,294	0·024	(0·034)	<0·001	0·38	0·36	0·63
	Memory Cognition	1,294	-0·058	(0·037)	0·003	0·52	0·93	0·60
**HBCS**	Global cognition	623	-0·033	(0·037)	0·75	0·47	0·65	0·65
	Memory Cognition	623	0·049	(0·013)	0·36	0·75	0·88	0·39
**NFBS66**	Global cognition	3,153	0·045	(0·013)	0·07	0·27	0·63	0·71
	Memory Cognition	3,153	0·011	(0·031)	0·07	0·27	0·63	0·71
**PIVUS**	Global cognition	672	0·018	(0·031)	0·01	0·06	0·06	0·50
	Memory Cognition	693	-0·140	(0·044)	0·01	0·04	0·06	0·18
**Tromsø**	Global cognition	2,032	-0·066	(0·044)	0·001	0·05	0·09	0·40
	Memory Cognition	2,151	0·022	(0·014)	0·96	0·30	0·58	0·44
**ULSAM**	Global cognition	829	0·027	(0·014)	0·61	0·38	-	0·45
**YFS**	Global Cognition	1,581	0·016	(0·009)	0·73	0·91	0·74	0·44
	Memory cognition	1,610	0·018	(0·009)	0·20	0·61	0·28	0·59

* Global cognition: $p_{heterogenity}$ = <0.001, I^2=78·56
Memory cognition: $p_{heterogenity}$ = <0.001, I^2=77·22

Supplementary Table 4. Metagression

	Meta regression for associations with global cognition				Meta regression for associations with memory cognition			
**Exposures**	***CYP2R1***	***DHCR7***	***Synthesis score***	**25(OH)D**	***CYP2R1***	***DHCR7***	***Synthesis score***	**25(OH)D**
**Study characteristic**	$\mathbf{P_{metaregression}}$	$\mathbf{P_{metaregression}}$	$\mathbf{P_{metaregression}}$	$\mathbf{P_{metaregression}}$	$\mathbf{P_{metaregression}}$	$\mathbf{P_{metaregression}}$	$\mathbf{P_{metaregression}}$	$\mathbf{P_{metaregression}}$
Age (% ≥65 years)	0·72	0·90	0·61	<0·001	0·05	0·73	0.13	0·05
Sex (% males)	0·58	0·80	0·91	0·91	0·49	0·51	0.28	0·75
Country (UK, Eurpoe, Nordic or US)	0·78	0·34	0·21	0·38	0·21	0·51	0.15	0·38
Vitamin D assay (mass spectrometry or immunoassay)	-	-	-	0·61	-	-	-	0·40

Supplementary Table 5. Association between genetic variants and cognitive function

Cohort	Outcome	Exposure	N	Coefficient, per vitamin D-decreasing allele	(se)	p-value
**Overall***	Global cognitive function	*DHCR7*	153,187	-0·0052	(0·0039)	0·18
		CYP2R1	153,187	0·0044	(0·0060)	0·46
		Synthesis score	153,187	-0·0003	(0·0043)	0·94
	Memory cognitive function	*DHCR7*	155,882	-0·0033	(0·0040)	0·40
		CYP2R1	155,882	0·0032	(0·0062)	0·61
		Synthesis score	155,882	-0·0022	(0·0035)	0·53
**1958BC**	Global cognitive function	*DHCR7*	4,808	-0·008	(0·02)	0·74
		CYP2R1	4,808	-0·004	(0·02)	0·82
		Synthesis score	4,808	-0·003	(0·02)	0·83
	Memory cognitive function	*DHCR7*	4,901	-0·007	(0·02)	0·76
		CYP2R1	4,901	-0·029	(0·02)	0·14
		Synthesis score	4,901	-0·018	(0·02)	0·23
**CoLaus**	Global cognitive function	*DHCR7*	866	0·033	(0·05)	0·52
		CYP2R1	866	0·054	(0·05)	0·24
		Synthesis score	866	0·043	(0·04)	0·23
**ESTHER**	Global cognitive function	*DHCR7*	1,331	-0·061	(0·04)	0·16
		CYP2R1	1,331	-0·029	(0·04)	0·44
		Synthesis score	1,331	-0·044	(0·03)	0·13
	Memory cognitive function	*DHCR7*	1,331	-0·036	(0·04)	0·41
		CYP2R1	1,331	-0·023	(0·04)	0·54
		Synthesis score	1,331	-0·030	(0·03)	0·30
**HBCS**	Global cognitive function	*DHCR7*	785	-0·007	(0·05)	0·89
		CYP2R1	785	0·040	(0·05)	0·42
		Synthesis score	785	0·013	(0·04)	0·73
	Memory cognitive function	*DHCR7*	785	-0·039	(0·05)	0·45
		CYP2R1	785	0·040	(0·05)	0·41
		Synthesis score	785	-0·002	(0·04)	0·96
**NFBC66**	Global cognitive function	*DHCR7*	3,328	-0·00004	(0·02)	1·00
		CYP2R1	3,328	0·017	(0·02)	0·49
		Synthesis score	3,328	0·012	(0·02)	0·52
	Memory cognitive function	*DHCR7*	3,328	-0·00004	(0·02)	1·00
		CYP2R1	3,328	0·017	(0·02)	0·49
		Synthesis score	3,328	0·012	(0·02)	0·52
**PIVUS**	Global cognitive function	*DHCR7*	676	0·020	(0·05)	0·70

		CYP2R1	676	-0·033	(0·05)	0·51
		Synthesis score	676	-0·013	(0·04)	0·73
	Memory cognitive function	*DHCR7*	697	0·004	(0·05)	0·93
		CYP2R1	697	-0·025	(0·05)	0·63
		Synthesis score	697	-0·020	(0·04)	0·61
**Tromsø**	Global cognitive function	*DHCR7*	2,253	0·011	(0·02)	0·63
		CYP2R1	2,253	0·064	(0·02)	0·01
		Synthesis score	2,253	0·042	(0·02)	0·02
	Memory cognitive function	*DHCR7*	2,385	-0·009	(0·03)	0·72
		CYP2R1	2,385	0·064	(0·03)	0·01
		Synthesis score	2,385	0·030	(0·02)	0·12
**ULSAM**	Global cognitive function	*DHCR7*	829	-0·029	(0·05)	0·53
		CYP2R1	829	-0·063	(0·05)	0·16
		Synthesis score	829	-0·050	(0·03)	0·15
**YFS**	Global cognitive function	*DHCR7*	1,587	0·013	(0·03)	0·70
		CYP2R1	1,587	0·014	(0·03)	0·68
		Synthesis score	1,587	0·011	(0·03)	0·68
	Memory cognitive function	*DHCR7*	1,616	-0·042	(0·03)	0·23
		CYP2R1	1,616	0·016	(0·04)	0·65
		Synthesis score	1,616	-0·019	(0·03)	0·46
**ASPS**	Global cognitive function	*DHCR7*	724	-0·072	(0·05)	0·11
		CYP2R1	724	-0·025	(0·04)	0·54
		Synthesis score	724	-0·051	(0·03)	0·10
	Memory cognitive function	*DHCR7*	763	-0·068	(0·05)	0·16
		CYP2R1	763	-0·025	(0·04)	0·56
		Synthesis score	763	-0·045	(0·03)	0·18
**ELSA**	Global cognitive function	*DHCR7*	5,270	-0·039	(0·02)	0·06
		CYP2R1	5,270	0·029	(0·02)	0·09
		Synthesis score	5,270	-0·002	(0·01)	0·87
	Memory cognitive function	*DHCR7*	5,382	-0·026	(0·02)	0·22
		CYP2R1	5,382	0·038	(0·02)	0·03
		Synthesis score	5,382	0·010	(0·01)	0·50
**HRS**	Global cognitive function	*DHCR7*	5,218	0·009	(0·02)	0·64
		CYP2R1	5,218	0·021	(0·01)	0·18
		Synthesis score	5,218	0·015	(0·01)	0·24
	Memory cognitive function	*DHCR7*	8,460	0·001	(0·01)	0·92
		CYP2R1	8,460	0·0003	(0·02)	0·99
		Synthesis score	8,460	0·001	(0·01)	0·90
**STR**	Global cognitive function	*DHCR7*	804	0·067	(0·04)	0·13

		CYP2R1	804	-0·030	(0·04)	0·48
		Synthesis score	804	0·010	(0·03)	0·76
	Memory cognitive function	*DHCR7*	893	0·047	(0·05)	0·31
		CYP2R1	893	0·018	(0·04)	0·69
		Synthesis score	893	0·028	(0·03)	0·41
**TwinGene**	Global cognitive function	*DHCR7*	2,322	-0·002	(0·030)	0·96
		CYP2R1	2,322	-0·019	(0·029)	0·50
		Synthesis score	2,322	-0·010	(0·022)	0·64
	Memory cognitive function	*DHCR7*	2,299	0·048	(0·031)	0·12
		CYP2R1	2,299	0·030	(0·029)	0·31
		Synthesis score	2,299	0·038	(0·023)	0·09
**UK Biobank**	Global cognitive function	*DHCR7*	109,911	-0·005	(0·005)	0·28
		CYP2R1	109,911	-0·001	(0·004)	0·73
		Synthesis score	109,911	-0·003	(0·003)	0·38
	Memory cognitive function	*DHCR7*	110,545	-0·001	(0·005)	0·86
		CYP2R1	110,545	-0·005	(0·004)	0·21
		Synthesis score	110,545	-0·003	(0·003)	0·29
**UKHLS**	Global cognitive function	*DHCR7*	8,480	-0·012	(0·02)	0·47
		CYP2R1	8,480	-0·020	(0·01)	0·14
		Synthesis score	8,480	-0·016	(0·01)	0·14
	Memory cognitive function	*DHCR7*	8,480	-0·018	(0·02)	0·27
		CYP2R1	8,480	-0·017	(0·01)	0·20
		Synthesis score	8,480	-0·016	(0·01)	0·12
**WII**	Global cognitive function	*DHCR7*	3,961	0·009	(0·02)	0·72
		CYP2R1	3,961	0·005	(0·02)	0·81
		Synthesis score	3,961	0·007	(0·02)	0·67
	Memory cognitive function	*DHCR7*	3,983	0·008	(0·03)	0·74
		CYP2R1	3,983	0·006	(0·02)	0·79
		Synthesis score	3,983	0·008	(0·02)	0·62

*Global cognition: *DHCR7* $p_{heterogenity}$=0·72, I^2=0; *CPY2R1* $p_{heterogenity}$ = 0·14, I^2=27·70; Synthesis score $p_{heterogenity}$ = 0·22, I^2=19·71.
Memory cognition: *DHCR7* $p_{heterogenity}$=0·68, I^2=0; *CPY2R1* $p_{heterogenity}$ = 0·15, I^2=27·27; Synthesis score $p_{heterogenity}$ = 0·.37, I^2=7·44

Supplementary Table 6: Association between genetic variants and 25(OH)D

Cohort	Genetic variant	N	Coefficient, change in 25(OH)D per vitamin D-decreasing allele*	(se)	p-value
**Overall***	*DHCR7*	18,830	-0·027	0·007	<0·001
	CYP2R1	18,830	-0·033	0·005	<0·001
	Synthesis score	18,830	-0·031	0·005	<0·001
**1958BC**	*DHCR7*	5,238	-0·054	0·010	<0·001
	CYP2R1	5,238	-0·034	0·008	<0·001
	Synthesis score	5,238	-0·043	0·006	<0·001
**CoLaus**	*DHCR7*	865	-0·098	0·027	<0·001
	CYP2R1	865	-0·063	0·024	0·01
	Synthesis score	865	-0·089	0·019	<0·001
**ESTHER**	*DHCR7*	1,362	-0·044	0·016	0·005
	CYP2R1	1,362	-0·007	0·014	0·594
	Synthesis score	1,362	-0·021	0·010	0·042
**HBCS**	*DHCR7*	707	0·006	0·017	0·731
	CYP2R1	707	-0·070	0·016	<0·001
	Synthesis score	707	-0·034	0·012	0·006
**NFBC66**	*DHCR7*	3,309	-0·022	0·006	0·001
	CYP2R1	3,309	-0·022	0·007	0·001
	Synthesis score	3,309	-0·023	0·005	<0·001
**PIVUS**	*DHCR7*	887	-0·015	0·016	0·358
	CYP2R1	887	-0·052	0·016	0·002
	Synthesis score	887	-0·034	0·012	0·006
**Tromsø**	*DHCR7*	3,321	-0·012	0·008	0·144
	CYP2R1	3,321	-0·020	0·009	0·019
	Synthesis score	3,321	-0·019	0·006	0·003
**ULSAM**	*DHCR7*	1,118	-0·015	0·013	0·269
	CYP2R1	1,118	-0·035	0·013	0·007
	Synthesis score	1,118	-0·023	0·010	0·017
**YFS**	*DHCR7*	2,023	-0·027	0·010	0·009
	CYP2R1	2,023	-0·035	0·010	0·001
	Synthesis score	2,023	-0·032	0·008	<0·001

DHCR7 $p_{heterogenity}$=0·002 , I^2=66·66; *CPY2R1* $p_{heterogenity}$ = 0·04, I^2=50·50; Synthesis score $p_{heterogenity}$ = 0·01, I^2=59·92

*Estimates were changed to reflect percentage change in 25(OH)D per vitamin D-decreasing allele[exp(estimate)-1*100][59]

Supplementary Table 7. Power calculation

Setting	Exposure	Parameter setting			Minimal effect size (Δ SD change in cognitive score per 1 SD change in ln(vitd))
		**Rsq**	**n**	**Power**	
Non-stratified analyses	*CYP2R1*	0·004	153,187	0·8	0·116
	DHCR7	0·003	153,187	0·8	0·134
	Synthesis score	0·006	153,187	0·8	0·092
Stratified analyses	*CYP2R1*	0·004	4,957	0·8	0·646
	DHCR7	0·003	4,957	0·8	0·743
	Synthesis score	0·006	4,957	0·8	0·514

A

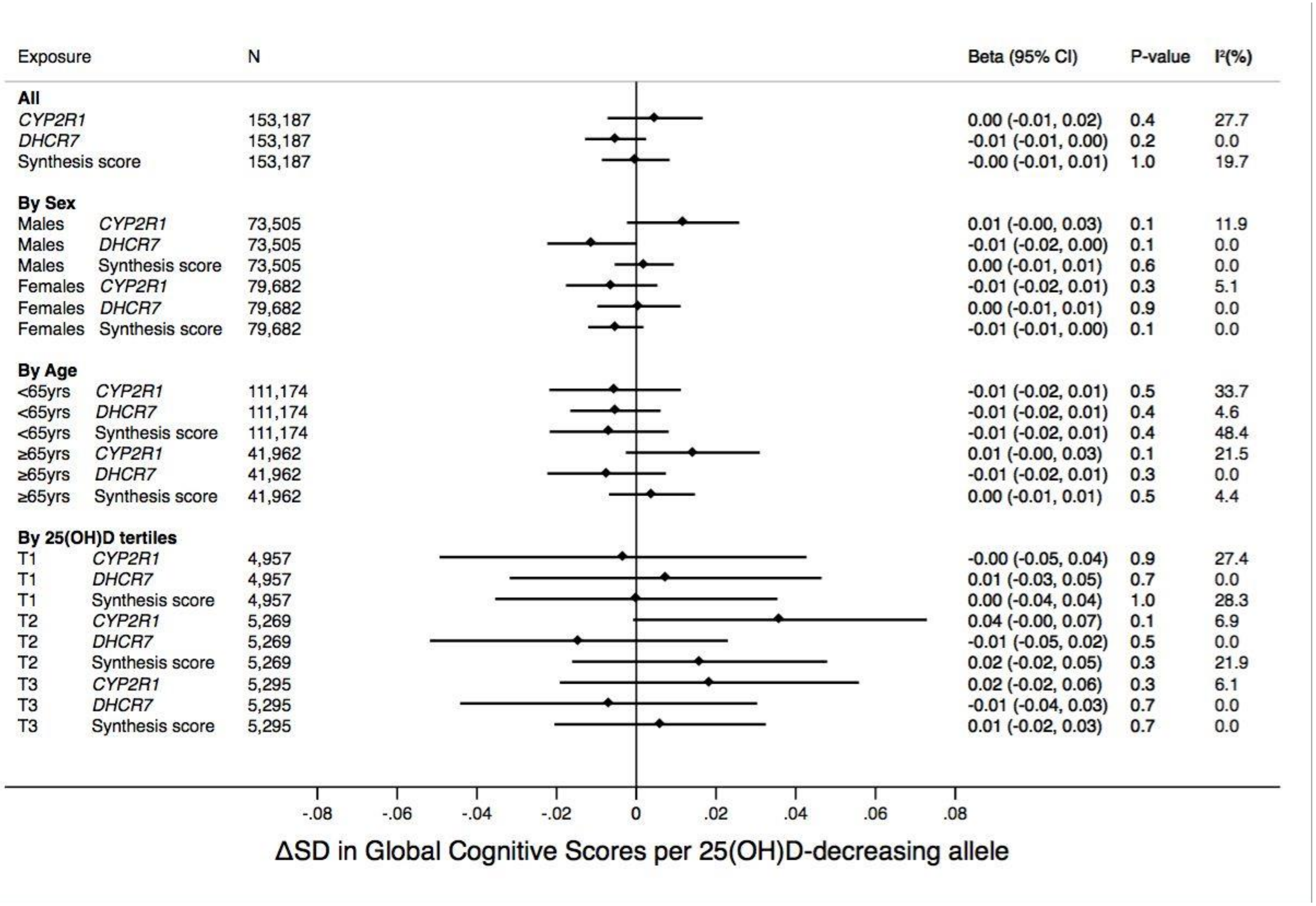
Exposure
N
Beta (95% CI)
P-value
I²(%)
All
CYP2R1 153,187 0.00 (-0.01, 0.02) 0.4 27.7
DHCR7 153,187 -0.01 (-0.01, 0.00) 0.2 0.0
Synthesis score 153,187 -0.00 (-0.01, 0.01) 1.0 19.7
By Sex
Males CYP2R1 73,505 0.01 (-0.00, 0.03) 0.1 11.9
Males DHCR7 73,505 -0.01 (-0.02, 0.00) 0.1 0.0
Males Synthesis score 73,505 0.00 (-0.01, 0.01) 0.6 0.0
Females CYP2R1 79,682 -0.01 (-0.02, 0.01) 0.3 5.1
Females DHCR7 79,682 0.00 (-0.01, 0.01) 0.9 0.0
Females Synthesis score 79,682 -0.01 (-0.01, 0.00) 0.1 0.0
By Age
<65yrs CYP2R1 111,174 -0.01 (-0.02, 0.01) 0.5 33.7
<65yrs DHCR7 111,174 -0.01 (-0.02, 0.01) 0.4 4.6
<65yrs Synthesis score 111,174 -0.01 (-0.02, 0.01) 0.4 48.4
≥65yrs CYP2R1 41,962 0.01 (-0.00, 0.03) 0.1 21.5
≥65yrs DHCR7 41,962 -0.01 (-0.02, 0.01) 0.3 0.0
≥65yrs Synthesis score 41,962 0.00 (-0.01, 0.01) 0.5 4.4
By 25(OH)D tertiles
T1 CYP2R1 4,957 -0.00 (-0.05, 0.04) 0.9 27.4
T1 DHCR7 4,957 0.01 (-0.03, 0.05) 0.7 0.0
T1 Synthesis score 4,957 0.00 (-0.04, 0.04) 1.0 28.3
T2 CYP2R1 5,269 0.04 (-0.00, 0.07) 0.1 6.9
T2 DHCR7 5,269 -0.01 (-0.05, 0.02) 0.5 0.0
T2 Synthesis score 5,269 0.02 (-0.02, 0.05) 0.3 21.9
T3 CYP2R1 5,295 0.02 (-0.02, 0.06) 0.3 6.1
T3 DHCR7 5,295 -0.01 (-0.04, 0.03) 0.7 0.0
T3 Synthesis score 5,295 0.01 (-0.02, 0.03) 0.7 0.0
-.08 -.06 -.04 -.02 0 .02 .04 .06 .08
ΔSD in Global Cognitive Scores per 25(OH)D-decreasing allele

B

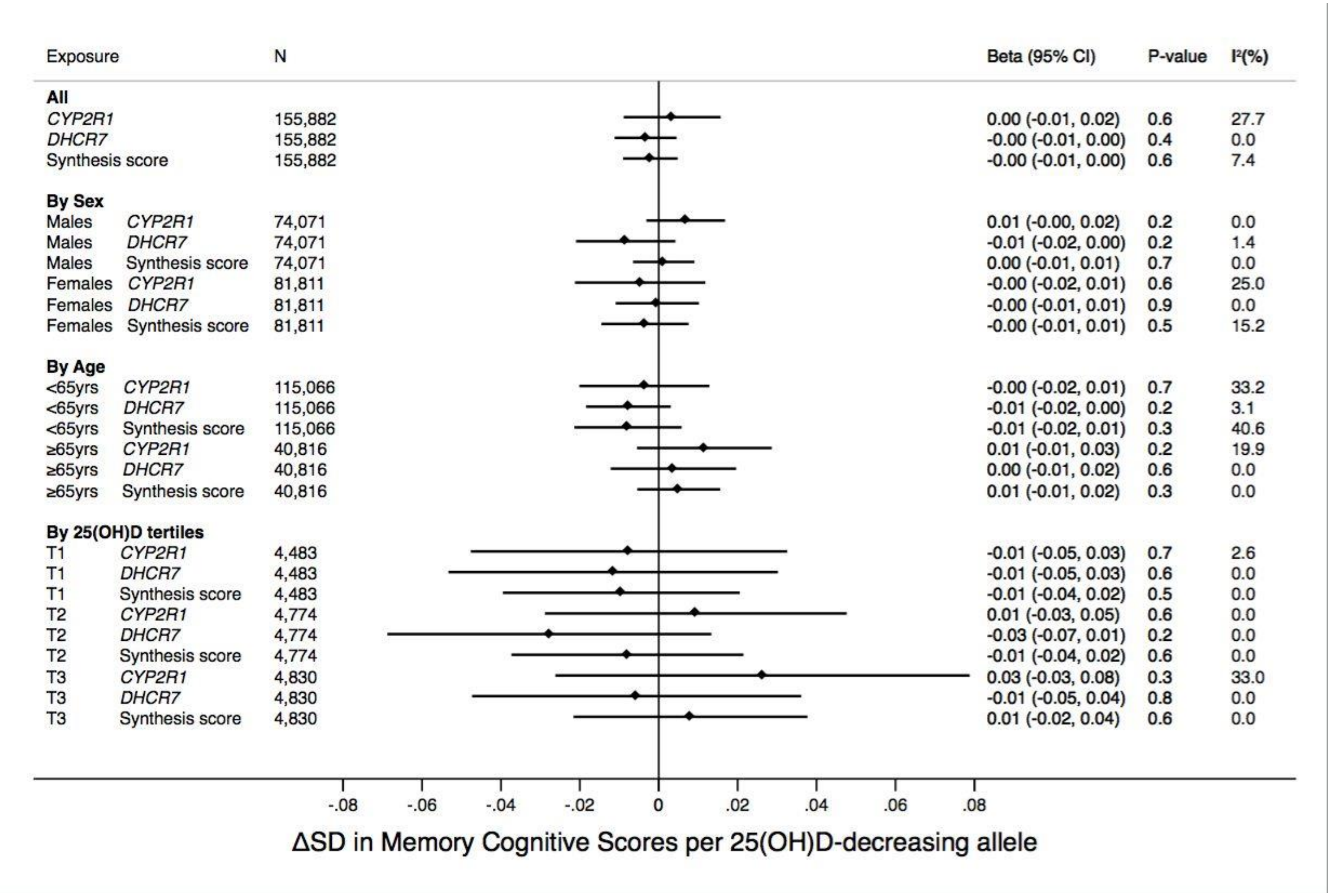

Supplementary figure 1. Stratified association of CYP2R1, DHCR7 and synthesis score with global cognition (A) and memory cognition (B)

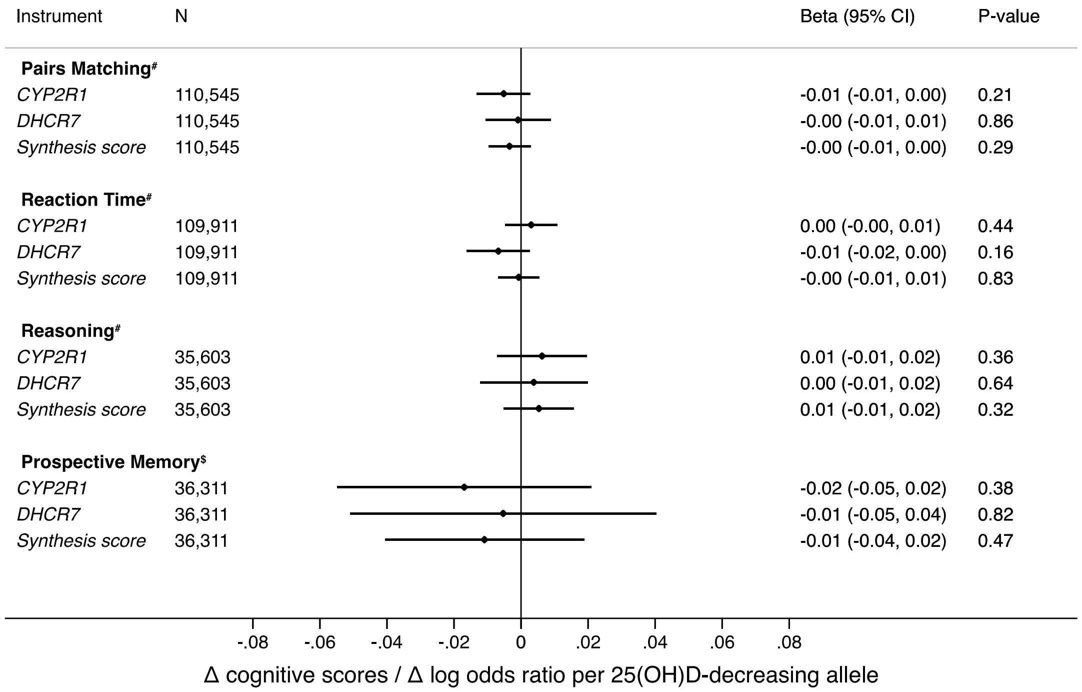

Supplementary figure 2. Vitamin D SNPs and cognitive domain-specific effects in UK Biobank;

#Effect size = a change in cognitive scores per 25(OH)D-decreasing allele; $Effect size = a change in log odds ratio per 25(OH)D-decreasing allele. For all cognitive tests, positive effect sizes indicate improvements in cognitive function. For the prospective memory (i.e. a binary outcome), odds = probability of being correct / probability of being incorrect at the first attempt.

Additional funding sources

Study-specific acknowledgments

We are grateful to all the participants in the 1958BC. We acknowledge Professor Ian Gibb, Dr Steve Turner, and Marie-Claude Fawcett (Royal Victoria Infirmary, Newcastle-upon-Tyne, U.K.) for carrying out the laboratory assays. This work made use of data and samples generated by the 1958 Birth Cohort (NCDS) , which is managed by the Centre for Longitudinal Studies at the UCL Institute of Education, funded by the Economic and Social Research Council (grant number ES/M001660/1). Access to these resources was enabled via the 58READIE Project funded by Wellcome Trust and Medical Research Council (grant numbers WT095219MA and G1001799). A full list of the financial, institutional and personal contributions to the development of the 1958 Birth Cohort Biomedical resource is available at http://www2.le.ac.uk/projects/birthcohort/1958bc/about/contributors-funders. The Medical Research Council funded the 2002–2004 clinical follow-up of the 1958 birth cohort (grant G0000934), 25(OH)D assays were funded by the BUPA Foundation. Further support was obtained from the UK Medical Research Council (grant G0601653 and SALVE/PREVMEDSYN with Academy of Finland). Genotyping was undertaken as part of the Wellcome Trust Case-Control Consortium (WTCCC) under Wellcome Trust award 076113, and a full list of the investigators who contributed to the generation of the data is available at www.wtccc.org.uk. This research used resources provided by the Type 1 Diabetes Genetics Consortium, a collaborative clinical study sponsored by the National Institute of Diabetes and Digestive and Kidney Diseases (NIDDK), National Institute of Allergy and Infectious diseases, National Human Genome Research Institute, National Institute of Child Health and Human Development, and Juvenile Diabetes Research Foundation International (JDRF) and supported by U01DK062418. Great Ormond Street Hospital/University College London, Institute of Child Health receives a proportion of funding from the Department of Health’s National Institute for Health Research (NIHR) (’Biomedical Research Centres’ funding. The UK Household Longitudinal Study is led by the Institute for Social and Economic Research at the University of Essex and funded by the Economic a Social Research Council (ES/F000537/1). The data were collected by NatCen and the genome wide scan data were analysed by the Wellcome Trust Sanger Institute. The ESTHER study was funded by the Baden-Württemberg state Ministry of Science, Research and Arts (Stuttgart, Germany), the Federal Ministry of Education and Research (Berlin, Germany) and the Federal Ministry of Family Affairs, Senior Citizens, Women and Youth (Berlin, Germany). Measurements of 25(OH)D in men were conducted in the context of the German Cancer Aid project number 108250 and 108426. The funders had no role in the design, conduct and reporting of this study. Samples from the English Longitudinal Study of Ageing DNA Repository (EDNAR), which receives support from the National Institute on Aging (NIA: R01 AG017644; R01AG1764406S1) and the Economic and Social Research Council (ESRC: ES/K005774/1), were used in this study. The TromsØ study recives support from UiT-The Arctic University of Norway, Norwegian Research Council, North Norway Health Authorities.

The Whitehall II study was supported by the UK Medical Research Council (K013351) and the US National Institutes of Health (HL036310, AG034454). The Young Finns Study has been financially supported by the Academy of Finland: grants 286284 (T.L.), 134309 (Eye), 126925, 121584, 124282, 129378 (Salve), 117797 (Gendi), and 41071 (Skidi), the Social Insurance Institution of Finland, Kuopio, Tampere and Turku University Hospital Medical Funds, Juho Vainio Foundation, Sigrid Juselius Foundation, Yrjö Jahnsson Foundation, Paavo Nurmi Foundation, Finnish Foundation for Cardiovascular Research, Finnish Cultural Foundation, Tampere Tuberculosis Foundation and Emil Aaltonen Foundation. STR/TwinGene is supported by National Institute of Aging (AG04563, AG10175), The MacArthur Foundation Research Network on Successful Aging, Swedish Research Council (825-2007-7460, 825-2009-6141, 825-3011-6182,521-2013-8689, 2015-03255), and the Swedish Council for Working Life and Social Research (FAS/FORTE) (97:0147:1B, 2009-0795,2013-2292).GENDER is supported by the MacArthur Foundation Research Network on Successful Aging, the Axel and Margaret Ax:son Johnsons Foundation, the Swedish Council for Social Research and the Swedish Foundation for Health Care Sciences and Allergy Research.

Personal funding statements

JM held a UCL Impact studentship with support from Jeans for Genes at the time of initiation and preliminary analyses of this study. EK acknowledges funding from the Mary Kinross Charitable Trust, the Halpin Trust and the James Tudor Foundation. DL acknowledges funding from the Mary Kinross Charitable Trust, the Halpin Trust, the James Tudor Foundation, the Rosetrees Trust, the Mason Foundation and the National Institute. HSchi acknowledges grants from The North Norway Regional Health Authority, The Norwegian Research Council, and UiT The Arctic University of Norway. RJ acknowledges grants from The North Norway Regional

Health Authority (151031/15082), The Norwegian Research Council (213787/F20), and UiT The Arctic University of Norway (311322 225109). MKi receives funding from UK Medical Research Council (k013351). EI receives support from NIH/NIDDK 1R01DK106236-01A1; NIH/NHLBI 1R01HL135313-01. AS received a postdoctoral fellowship grant from the Liaison Committee between the Central Norway Regional Health Authority and the Norwegian University of Science and Technology.

References

1. Power C, Elliott J. Cohort profile: 1958 British birth cohort (National Child Development Study). International Journal of Epidemiology. 2006;35(1):34-41.
2. Ofstedal MB, Fisher GG, Herzog AR. Documentation of Cogntive Functioning Measures in the Health and Retirement Study. 2005. Contract No.: HRS Documentation Report DR-006.
3. Roth M. CAMDEX-R: the Cambridge examination for mental disorders of the elderly: Cambridge University Press; 1998.
4. Richards M, Kuh D, Hardy R, Wadsworth M. Lifetime cognitive function and timing of the natural menopause. Neurology. 1999;53(2):308.
5. Burton PR, Clayton DG, Cardon LR, Craddock N, Deloukas P, Duncanson A, et al. Genome-wide association study of 14,000 cases of seven common diseases and 3,000 shared controls. Nature. 2007;447(7145):661-78.
6. Sawcer S, Hellenthal G, Pirinen M, Spencer CC, Patsopoulos NA, Moutsianas L, et al. Genetic risk and a primary role for cell-mediated immune mechanisms in multiple sclerosis. Nature. 2011;476(7359):214.
7. Craddock N, Hurles ME, Cardin N, Pearson RD, Plagnol V, Robson S, et al. Genome-wide association study of CNVs in 16,000 cases of eight common diseases and 3,000 shared controls. Nature. 2010;464(7289):713-20.
8. Barrett JC, Clayton DG, Concannon P, Akolkar B, Cooper JD, Erlich HA, et al. Genome-wide association study and meta-analysis find that over 40 loci affect risk of type 1 diabetes. Nature genetics. 2009;41(6):703-7.
9. Todd JA, Walker NM, Cooper JD, Smyth DJ, Downes K, Plagnol V, et al. Robust associations of four new chromosome regions from genome-wide analyses of type 1 diabetes. Nature genetics. 2007;39(7):857-64.
10. Goldberg DP, Cooper B, Eastwood MR, Kedward HB, Shepherd M. A standardized psychiatric interview for use in community surveys. British Journal of Preventive & Social Medicine. 1970;24(1):18-23.
11. Lewis G, Pelosi AJ, Araya R, Dunn G. Measuring psychiatric disorder in the community: a standardized assessment for use by lay interviewers. Psychological Medicine. 1992;22(2):465-86.
12. Firmann M, Mayor V, Vidal PM, Bochud M, Pecoud A, Hayoz D, et al. The CoLaus study: a population-based study to investigate the epidemiology and genetic determinants of cardiovascular risk factors and metabolic syndrome. BMC Cardiovasc Disord. 2008;8:6.
13. Bruce SJ, Rochat B, Beguin A, Pesse B, Guessous I, Boulat O, et al. Analysis and quantification of vitamin D metabolites in serum by ultra-performance liquid chromatography coupled to tandem mass spectrometry and high-resolution mass spectrometry--a method comparison and validation. Rapid communications in mass spectrometry : RCM. 2013;27(1):200-6.
14. Folstein MF, Folstein SE, McHugh PR. Mini-Mental State: a practical method for grading the cognitive state of patients for the clinician: Pergamon Press; 1975.
15. Nurnberger JI, Jr., Blehar MC, Kaufmann CA, York-Cooler C, Simpson SG, Harkavy-Friedman J, et al. Diagnostic interview for genetic studies. Rationale, unique

features, and training. NIMH Genetics Initiative. Arch Gen Psychiatry. 1994;51(11):849-59; discussion 63-4.
16. Breitling LP, Wolf M, Müller H, Raum E, Kliegel M, Brenner H. Large-scale application of a telephone-based test of cognitive functioning in older adults. Dementia and Geriatric Cognitive Disorders. 2010;30(4):309-16.
17. Raum E, Rothenbacher D, Löw M, Stegmaier C, Ziegler H, Brenner H. Changes of cardiovascular risk factors and their implications in subsequent birth cohorts of older adults in Germany: a life course approach. European Journal of Cardiovascular Prevention & Rehabilitation. 2007;14(6):809-14.
18. Schöttker B, Haug U, Schomburg L, Köhrle J, Perna L, Müller H, et al. Strong associations of 25-hydroxyvitamin D levels with all-cause, cardiovascular, cancer and respiratory disease mortality in a large cohort study. Experimental and Clinical Endocrinology & Diabetes. 2013;121(03):P95.
19. Schöttker B, Jansen EH, Haug U, Schomburg L, Köhrle J, Brenner H. Standardization of misleading immunoassay based 25-hydroxyvitamin D levels with liquid chromatography tandem-mass spectrometry in a large cohort study. PloS one. 2012;7(11):e48774.
20. Wechsler D. Wechsler Memory Scale, Revised Manual. San Antiono, TX: Psychologicla Corp; 1987.
21. Wechsler D. Wechsler Adult Intelligence Scale, Revised Manual. San Antonin, TX: Psychological Corp; 1981.
22. Benton, Hamsher. Multilingual aphasia examination. Iowa city: University of Iowa Press; 1976.
23. Yesavage JA, Brink T, Rose TL, Lum O, Huang V, Adey M, et al. Development and validation of a geriatric depression screening scale: a preliminary report. Journal of Psychiatric Research. 1983;17(1):37-49.
24. Barker DJ, Osmond C, Forsén TJ, Kajantie E, Eriksson JG. Trajectories of growth among children who have coronary events as adults. New England Journal of Medicine. 2005;353(17):1802-9.
25. Rantakallio P. Groups at risk in low birth weight infants and perinatal mortality. Acta Paediatrica Scandinavica. 1969;193:Suppl 193: 1+.
26. Williams DM, Palaniswamy S, Sebert S, Buxton JL, Blakemore AI, Hyppönen E, et al. 25-Hydroxyvitamin D Concentration and Leukocyte Telomere Length in Young Adults: Findings From the Northern Finland Birth Cohort 1966. American journal of epidemiology. 2016;183(3):191-8.
27. Solomon PR, Hirschoff A, Kelly B, Relin M, Brush M, DeVeaux RD, et al. A 7 minute neurocognitive screening battery highly sensitive to Alzheimer's disease. Archives of Neurology. 1998;55(3):349.
28. Dawodu A, Agarwal M, Hossain M, Kochiyil J, Zayed R. Hypovitaminosis D and vitamin D deficiency in exclusively breast-feeding infants and their mothers in summer: a justification for vitamin D supplementation of breast-feeding infants. The Journal of pediatrics. 2003;142(2):169-73.
29. Jacobsen BK, Eggen AE, Mathiesen EB, Wilsgaard T, Njølstad I. Cohort profile: the Tromsø study. International Journal of Epidemiology. 2012;41(4):961-7.
30. Grimnes G, Almaas B, Eggen AE, Emaus N, Figenschau Y, Hopstock LA, et al. Effect of smoking on the serum levels of 25-hydroxyvitamin D depends on the assay employed. European journal of endocrinology. 2010;163(2):339-48.
31. Arntzen K, Schirmer H, Wilsgaard T, Mathiesen E. Impact of cardiovascular risk factors on cognitive function: the Tromsø study. European Journal of Neurology. 2011;18(5):737-43.

32. Derogatis LR, Lipman RS, Rickels K, Uhlenhuth EH, Covi L. The Hopkins Symptom Checklist (HSCL): A self-report symptom inventory. Behavioral Science. 1974;19(1):1-15.
33. Michaëlsson K, Baron JA, Snellman G, Gedeborg R, Byberg L, Sundström J, et al. Plasma vitamin D and mortality in older men: a community-based prospective cohort study. The American journal of clinical nutrition. 2010;92(4):841-8.
34. Uppsala Universitet. ULSAM-70 [Available from: http://www2.pubcare.uu.se/ULSAM/invest/70yrs/meth70.htm#15.
35. World Health Organisation. International Classification of Diseases (ICD) 2010 [Available from: http://www.who.int/classifications/icd/en/.
36. Raitakari OT, Juonala M, Rönnemaa T, Keltikangas-Järvinen L, Räsänen L, Pietikäinen M, et al. Cohort profile: the cardiovascular risk in Young Finns Study. International journal of epidemiology. 2008;37(6):1220-6.
37. Juonala M, Voipio A, Pahkala K, Viikari JS, Mikkilä V, Kähönen M, et al. Childhood 25-OH vitamin D levels and carotid intima-media thickness in adulthood: the cardiovascular risk in young Finns study. The Journal of Clinical Endocrinology & Metabolism. 2015;100(4):1469-76.
38. Seppälä I, Kleber ME, Lyytikäinen L-P, Hernesniemi JA, Mäkelä K-M, Oksala N, et al. Genome-wide association study on dimethylarginines reveals novel AGXT2 variants associated with heart rate variability but not with overall mortality. European heart journal. 2014;35(8):524-31.
39. Schmidt R, Lechner H, Fazekas F, Niederkorn K, Reinhart B, Grieshofer P, et al. Assessment of cerebrovascular risk profiles in healthy persons: definition of research goals and the Austrian Stroke Prevention Study (ASPS). Neuroepidemiology. 1994;13(6):308-13.
40. Schmidt R, Fazekas F, Kapeller P, Schmidt H, Hartung H-P. MRI white matter hyperintensities Three-year follow-up of the Austrian Stroke Prevention Study. Neurology. 1999;53(1):132-.
41. Janke W, Debus G. Die Eigenschaftswörterliste EWL: Verlag für Psychologie; 1978.
42. Steptoe A, Breeze E, Banks J, Nazroo J. Cohort Profile: The English Longitudinal Study of Ageing. International Journal of Epidemiology. 2012.
43. Radloff LS. The CES-D scale a self-report depression scale for research in the general population. Applied psychological measurement. 1977;1(3):385-401.
44. Documentation of Affective Functioning Measures the Health and Retirement StudyMi. University of Michigan: Survey, Research Centre,; 2000. Contract No.: Report DR-005.
45. Juster FT, Suzman R. An overview of the Health and Retirement Study. Journal of Human Resources. 1995:S7-S56.
46. Zivin K, Llewellyn DJ, Lang IA, Vijan S, Kabeto MU, Miller EM, et al. Depression among older adults in the United States and England. The American Journal of Geriatric Psychiatry. 2010;18(11):1036-44.
47. Lichtenstein P, Floderus B, Svartengren M, Svedberg P, Pedersen NL. The Swedish Twin Registry: a unique resource for clinical, epidemiological and genetic studies. Journal of internal medicine. 2002;252(3):184-205.
48. Pedersen NL, McClearn GE, Plomin R, Nesselroade JR, Berg S, DeFaire U. The Swedish adoption twin study of aging: an update. Acta Geneticae Medicae et Gemellologiae: Twin Research. 1991;40(01):7-20.
49. Gold CH, Malmberg B, McClearn GE, Pedersen NL, Berg S. Gender and health a study of older unlike-sex twins. The Journals of Gerontology Series B: Psychological Sciences and Social Sciences. 2002;57(3):S168-S76.

50. Voight BF, Kang HM, Ding J, Palmer CD, Sidore C, Chines PS, et al. The metabochip, a custom genotyping array for genetic studies of metabolic, cardiovascular, and anthropometric traits. PLoS Genet. 2012;8(8):e1002793.
51. Magnusson PK, Almqvist C, Rahman I, Ganna A, Viktorin A, Walum H, et al. The Swedish Twin Registry: establishment of a biobank and other recent developments. Twin Research and Human Genetics. 2013;16(01):317-29.
52. McFall S, Petersen J, Kaminska O, Lynn P. Understanding Society The UK Household Longitudinal Study Waves 2 and 3 Nurse Health Assessment, 2010 – 2012 Guide to Nurse Health Assessment. Colchester: Institute for Social and Economic Research, University of Essex; 2014.
53. Marmot M, Brunner E. Cohort profile: the Whitehall II study. International Journal of Epidemiology. 2005;34(2):251-6.
54. Singh-Manoux A, Kivimaki M, Glymour MM, Elbaz A, Berr C, Ebmeier KP, et al. Timing of onset of cognitive decline: results from Whitehall II prospective cohort study. BMJ: British Medical Journal. 2012;344.
55. Heim A. AH 4 group test of general Intelligence. Windsor, UK: NFER-Nelson Publishing Company Ltd; 1970.
56. Raven J. Guide to using the Mill Hill vocabulary test with progressive matrices. London, UK: HK Lewis. 1965.
57. Borkowski JG, Benton AL, Spreen O. Word fluency and brain damage. Neuropsychologia. 1967;5(2):135-40.
58. Goldberg DP, Hillier V. A scaled version of the General Health Questionnaire. Psychological Medicine. 1979;9(01):139-45.
59. Cornell Statistical Consulting Unit. Interpreting Coefficients in Regression with Log-Transformed Variables. In: University C, editor. US2012.
